# Nanotechnology-donated ischemic stroke therapeutics: evolving strategies from the basic to the cutting-edge

**DOI:** 10.7150/thno.127504

**Published:** 2026-01-01

**Authors:** Yuting Wang, Jiaxu Xu, Wenxuan Yan, Shenwu Zhang, Cong Luo, Yuequan Wang

**Affiliations:** 1 Department of Pharmaceutics, Wuya College of Innovation, Shenyang Pharmaceutical University, Shenyang 110016, PR China.; 2 Joint International Research Laboratory of Intelligent Drug Delivery Systems of Ministry of Education, Shenyang Pharmaceutical University, Shenyang 110016, PR China.

**Keywords:** drug delivery, biomimetic nanotherapeutics, exosome, ischemic stroke

## Abstract

Ischemic stroke (IS) is accompanied by high disability and mortality. Thrombolysis and neuroprotection are the predominant therapeutic strategies for IS. However, thrombolytic drugs commonly suffer from hemorrhagic risks and unsatisfactory thrombus-targeting delivery. Additionally, the blood-brain barrier (BBB) presents a significant challenge for the effective delivery of neuroprotective drugs. In recent years, nanodrug delivery systems (nano-DDS) have garnered significant attention for their ability to improve drug efficacy *in vivo* and facilitate BBB penetration. Specifically, thrombus- and cerebral ischemic lesion-targeted nano-DDS have emerged as a versatile toolbox for the precise treatment of IS. Herein, this paper provides an overview on the latest advancements in nano-DDS for IS therapy, covering conventional nanomedicines, cell membrane-camouflaged biomimetic nano-DDS, and exosome-involved nanotherapeutics, with a particular focus on the potential and application of cutting-edge nano-drug delivery techniques. Finally, we discuss the future perspectives and challenges of nano-DDS in the context of IS treatment.

## 1. Introduction

Stroke represents a leading global cause of mortality and permanent disability, with ischemic stroke (IS) constituting approximately 80% of all cases 1. According to the latest data from the global burden of disease study, IS remains a devastating global health crisis, responsible for an estimated 7.80 million new cases and 3.59 million deaths worldwide in 2021 2. Despite advances in therapeutic interventions, thrombolytic therapy remains the standard clinical treatment for IS, with tissue plasminogen activator (tPA) being the only FDA-approved thrombolytic agent 3, 4. However, the clinical application of tPA is constrained by several challenges, including a narrow therapeutic window (≤4.5 h), a high risk of hemorrhagic transformation and other complications, a short half-life (4-8 min), and limited thrombus affinity 5-7. Furthermore, while some antiplatelet agents and anticoagulants can promote microvascular vasodilation and reperfusion, their high doses may increase the risk of gastrointestinal and intracranial bleeding 8.

The pathophysiology of IS is complex, involving a cascade of events that lead to neuronal death. Initially, the ischemic and hypoxic environment prevents neurons from maintaining normal physiological homeostasis 9, 10, triggering the following processes: (i) excitotoxicity, primarily due to excess glutamate production, a neurotoxic excitatory neurotransmitter; (ii) oxidative and nitrosative stress, driven by an overproduction of reactive oxygen species and reactive nitrogen species (RONS), coupled with insufficient antioxidant defense during ischemia, leading to apoptotic cell death; and (iii) inflammatory response, mainly attributed to microglia, which are the brain's resident macrophages and are highly activated after brain injury, resulting in the production of substantial amounts of pro-inflammatory cytokines, as well as toxic metabolites and enzymes. Among these, the inflammatory response is particularly critical in the pathogenesis of IS. These pathophysiological processes cause substantial neuronal damage and exacerbate disease progression 9. In addition, the recanalization of blood flow by thrombolytic therapy may also provoke further oxidative stress due to the overexpression of RONS, which further accelerates brain tissue damage (namely, ischemia-reperfusion injury) 11. These point to a major role for the application of neuroprotective agents such as excitotoxicity inhibitors, antioxidants, and anti-inflammatory agents during or after thrombolysis to improve the pathological microenvironment and promote neurological recovery 9, 10, 12, 13. Unfortunately, these neuroprotective agents are hindered by the difficult penetration of the blood-brain barrier (BBB), poor water solubility and short half-life, resulting in suboptimal therapeutic efficacy 13-15.

**Figure 1:**
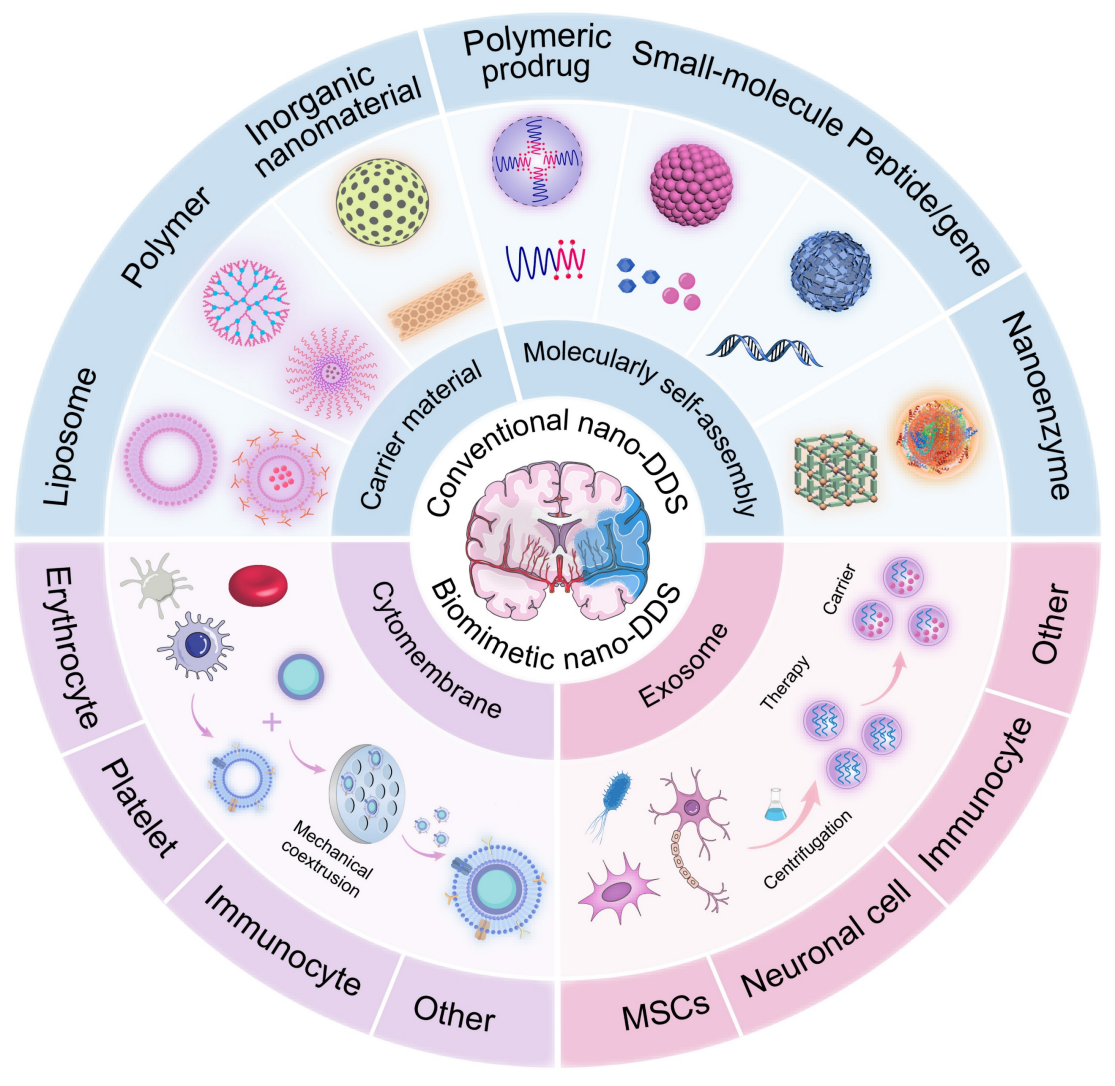
Schematic representation of nanotherapeutics for the treatment of IS.

Taken together, there is an urgent need to develop a novel drug delivery system to specifically deliver drugs to the site of thrombosis and brain tissue injury. In recent years, nanodrug delivery systems (nano-DDS) have been demonstrated as promising platforms for the treatment of IS with the following advantages: (i) improved water solubility and extended systemic circulation time of drugs, reducing the required drug dose; (ii) enhanced ability to cross the BBB; (iii) targeted delivery to the brain, facilitated by surface modifications of nanoparticles or cell membrane camouflage, resulting in a lower risk of off-targeting effects; and (iv) the potential for multi-drug delivery, enabling the simultaneous targeting of multiple therapeutic pathways. This review will provide an overview of current advances in the application of nano-DDS for IS, including conventional nano-DDS, cytomembrane-camouflaged biomimetic nanomedicines and exosome-involved nanotherapeutics. Moreover, we will detail the advantages and limitations of conventional nano-DDS, with special emphasis on the potential and application of novel biomimetic nano-DDS in the treatment of IS (**Figure 1**).

## 2. Pathophysiological features of IS and implications for nanotherapy

**Figure 2:**
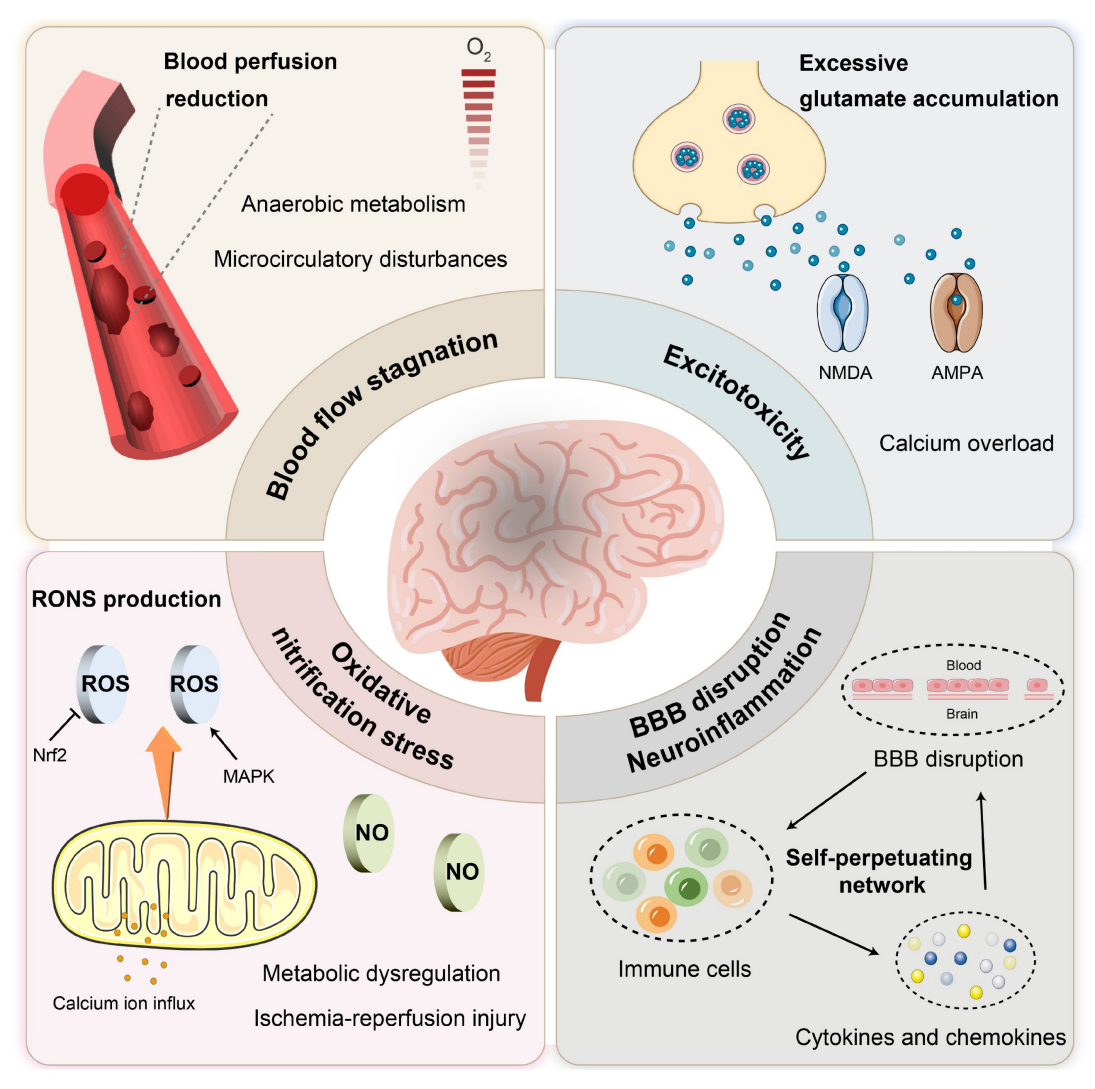
Schematic diagram of mechanisms related to pathological processes in IS.

**Figure 3:**
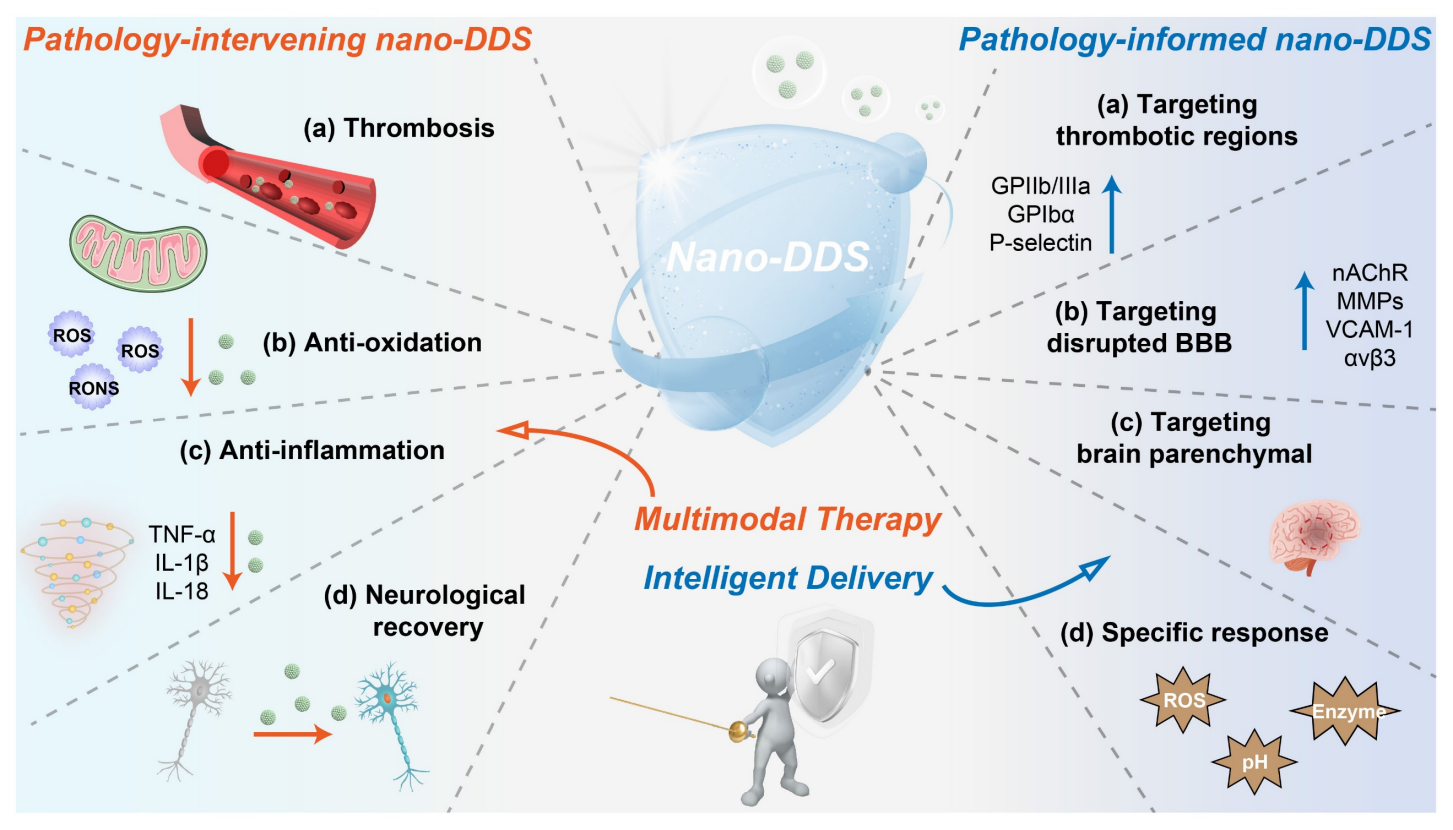
Integrative schematic linking pathophysiological mechanisms to nano-DDS interventions in IS.

IS results from a sudden reduction in cerebral blood flow, leading to hypoxia, nutrient deprivation, and ultimately neuronal death. As an acute and severe neurological event, IS involves multiple interconnected pathological processes that collectively drive tissue injury. This section outlines key pathophysiological mechanisms involved in IS, including cerebral ischemia 16, 17, excitotoxicity 18, BBB disruption 19, 20, inflammation and oxidative and nitrosative stress 21, 22, with an emphasis on aspects most relevant to the rational design of nano-DDS (**Figure 2** and **Figure 3**). These insights provide a pathophysiological foundation for nanotechnology-based interventions discussed in subsequent sections.

### 2.1 Blood flow stagnation

Cerebral arterial occlusion precipitates a sudden reduction in blood flow, leading to profound hypoxia and disruption of energy metabolism. Under ischemic conditions, cells shift to anaerobic glycolysis, leading to lactic acid accumulation and intracellular acidosis 23. This metabolic crisis is compounded by microcirculatory failure, which further limits oxygen delivery and exacerbates ischemic damage 24. The microvascular dysfunction not only amplifies initial injury but also complicates drug delivery, highlighting the need for nanocarriers capable of penetrating poorly perfused regions.

### 2.2 Excitotoxicity

Ischemia-induced energy depletion triggers neuronal depolarization and excessive release of glutamate, which overactivates N-methyl-D-aspartate (NMDA) and α-amino-3-hydroxy-5-methyl-4-isoxazolepropionic acid (AMPA) receptors. This leads to pathological calcium influx, activating degradative enzymes and generating oxidative stress 25. While excitotoxicity represents a well-established injury mechanism, its spatial and temporal progression offers opportunities for targeted nanotherapeutic intervention, particularly through receptors and ion channels enriched in ischemic regions.

### 2.3 BBB disruption and neuroinflammation

The disruption of the BBB is a hallmark of IS pathology. Ischemia impairs endothelial cell function and leads to the degradation of tight junction proteins (e.g., occludin, claudin, zonula occludens-1 (ZO-1)), thereby increasing paracellular permeability. This loss of barrier integrity allows the infiltration of leukocytes and plasma proteins, resulting in vasogenic edema and the activation of an inflammatory response. Furthermore, pericyte dysfunction exacerbates BBB breakdown, facilitating the entry of neurotoxic substances into the brain parenchyma 26.

BBB breakdown and subsequent immune cell recruitment activate a pro-inflammatory cascade. Microglia and astrocytes release cytokines such as tumor necrosis factor-α (TNF-α) and interleukin-1β (IL-1β), which enhance nuclear factor kappa-B (NF-κB) signaling and upregulate matrix metalloproteinase-9 (MMP-9) expression. Elevated MMP-9 further degrades tight junction proteins, establishing a self-perpetuating cycle of BBB impairment and inflammation 22, 27, 28, 29. The dynamic interplay between BBB integrity and immune response provides multiple targeting opportunities for nano-based strategies aimed at restoring barrier function and modulating inflammation.

### 2.4 Oxidative and nitrosative stress

Ischemia-reperfusion induces massive production of RONS, overwhelming endogenous antioxidant defenses 21, 30. The nuclear factor erythroid derived 2-like 2 (Nrf2), which plays a crucial role in regulating antioxidant gene expression, is often impaired following stroke, thereby reducing cellular defenses against oxidative damage. Furthermore, mitochondrial dysfunction during reperfusion exacerbates ROS generation and calcium overload, contributing to secondary neuronal injury 31, 32. These processes represent critical targets for nanotherapies, which can mitigate oxidative stress and reduce reperfusion-induced damage.

Thrombolytic agents such as tPA represent the standard of care for IS. However, thrombolytic therapy remains clinically limited by several factors, including time window restrictions, side effects, and variable efficacy among different patient populations. Despite the successful restoration of blood flow through revascularization, ischemia-reperfusion injury may paradoxically exacerbate cerebral damage. The multifaceted pathophysiology discussed earlier, encompassing excitotoxicity, neuroinflammation, oxidative stress, and disruption of the BBB, highlights the urgent need for adjunctive neuroprotective strategies (such as excitotoxicity inhibitors, antioxidants, and anti-inflammatory agents) that target these secondary injury mechanisms. Although numerous therapeutic candidates have shown promise in preclinical studies, their clinical translation has been hampered by intrinsic limitations, including poor bioavailability, rapid clearance, and most critically, inadequate penetration across the BBB. Fortunately, the emergence of nanotechnology has paved the way for novel approaches in IS treatment 33, 34, revealing significant potential with the advantages of enhanced targeting, increased drug payload, controlled release, and reduced side effects (**Figure 3**).

## 3. The emergence and evolution of conventional nanomedicines

Drug delivery across the BBB remains a major challenge in treating IS 35. However, recent evidence suggests that using nano-DDS can be an effective approach to overcome this barrier 36-41. Nanodrugs can cross the BBB through various transcellular pathways 42, 43. It is well-established that the BBB in the ischemic zone becomes dysfunctional following IS, with increased membrane permeability, which theoretically allows drugs to enter the brain parenchyma. However, the extent of BBB disruption is often insufficient to allow efficient nanodrugs entry into the brain. Interestingly, the disrupted BBB in the ischemic region can express specific proteins such as CXCR4, nicotinic acetylcholine receptor (nAChR), integrin αvβ3, MMPs, vascular cell adhesion molecule-1 (VCAM-1), and cannabinoid receptor 1 (CB1). These receptors can mediate the penetration of nanodrugs into the ischemic zone via interactions with specific targeting molecules. Additionally, thrombotic regions also exhibit overexpression of proteins like glycoprotein IIb/IIIa (GPIIb/IIIa), glycoprotein Ibα (GPIbα), and P-selectin, which can further facilitate targeted delivery of nanodrugs 14. Therefore, nano-DDS modified with specific chemical targeting blocks can precisely target brain infarction sites by effectively penetrating BBB or thrombus sites.

The ischemic environment in IS is characterized by several distinctive features compared to normal physiological conditions 44. First, the pH in the ischemic zone drops from a typical 7.35 to around 6.5 due to oxygen deprivation and increased lactic acid accumulation 45, 46. Second, elevated levels of ROS are found at both thrombotic and ischemic sites, playing a key role in mediating the associated inflammatory responses 47-49. Furthermore, enzymes such as thrombin and MMP-9 are also upregulated in these regions 50. These pathological microenvironments can serve as sensitive triggers for controlled drug release, enabling the selective targeting of ischemic tissues, thereby improving therapeutic efficacy and reducing side effects (**Figure 3**).

Given the unique advantages of nanodrugs in IS treatment, this section will focus on conventional nano-DDS, including liposome, polymeric nanoparticles, inorganic nanoparticles, carrier-free self-assembly nanoassemblies and multifunctional nanozymes. We will discuss their respective advantages and limitations in the context of drug delivery to the brain, with an emphasis on their potential for overcoming the challenges associated with the BBB. Additionally, we will explore the future trends and developments in these nanosystems, examining how they might evolve to enhance therapeutic outcomes in IS treatment. The recently developed conventional nano-DDS are summarized in **Table 1** and **Table 2**.

### 3.1. Liposomes

**Figure 4:**
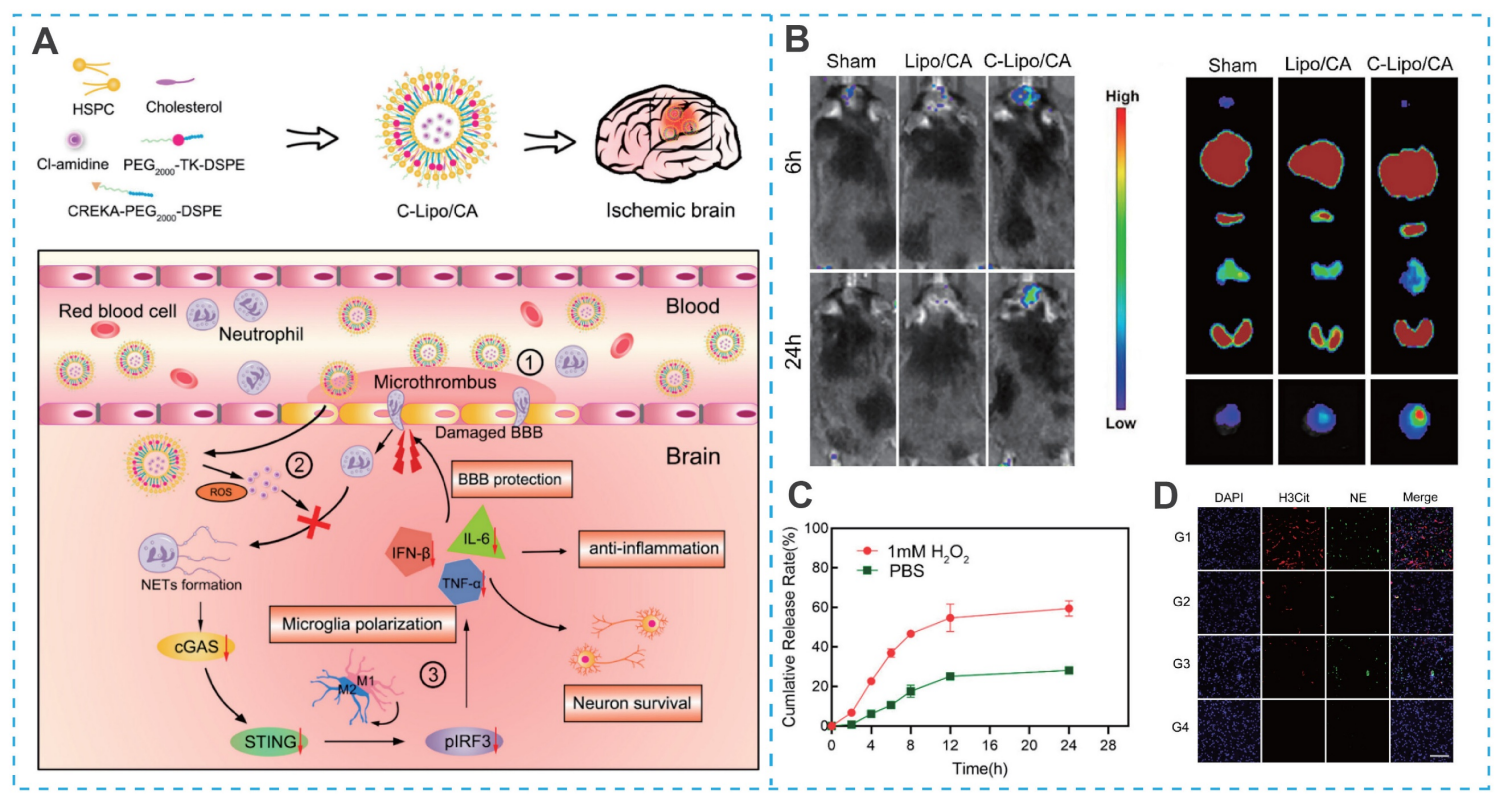
** Liposome-based nano-DDS for IS treatment. (A)** Illustration of the construction of C-Lipo/CA liposomes. (B) Thrombus targeting ability and in vivo distribution of C-Lipo/CA. (C) Cumulative release of Cl-amidine from C-Lipo/CA with or without 1 mM H_2_O_2_. (D) NETs regulation and BBB protection of C-Lipo/CA *in vivo*. Reproduced, with permission, from 60. Copyright (2023), American Chemical Society.

As spherical vesicles formed from natural phospholipids, liposomes constitute one of the most mature and extensively studied nanocarrier systems 35, 51, 52. Liposome-based drug delivery systems offer extended blood circulation times, favorable biodistribution, and significantly reduced systemic toxicity, while also being amenable to large-scale production 53-56. These advantages have facilitated their clinical translation in oncology. However, the application of liposomes for IS therapy remains limited, primarily due to their poor brain biodistribution, which severely compromises their therapeutic potential 35. As a result, enhancing brain targeting via surface functionalization has become a key focus in the field 57-59. For example, Sun *et al*. developed a brain-targeted, ROS-responsive liposome (C-Lipo/CA) for the efficient delivery of Cl-amidine (a peptidylarginine deiminase 4 (PAD4) inhibitor), utilizing CREKA (Cys-Arg-Glu-Lys-Ala)-PEG_2000_-DSPE and PEG_2000_-thioketone (TK)-DSPE (**Figure 4A**) 60. The CREKA peptide facilitated liposome crossing of BBB and targeted ischemic lesions by binding to fibronectin in microthrombi (**Figure 4B**). The ROS-sensitive TK bond triggered controlled Cl-amidine release at the ischemic site, minimizing off-target toxicity (**Figure 4C**). In a mouse model of cerebral artery occlusion/reperfusion (MCAO), the C-Lipo/CA mitigated ischemia-reperfusion injury by polarizing microglia toward an M2 phenotype and preserving BBB integrity (**Figure 4D**) 68.

Beyond active targeting, Zahraa S. Al-Ahmady *et al.* found that the time window of liposome administration also affected the brain biodistribution 61. This time-dependent selective distribution correlated with changes in BBB permeability. Their study showed that liposomes were more readily distributed in the neurovascular regions in the early hours after stroke, whereas 48 h post-stroke, liposome uptake was selectively enhanced in inflammatory cells, particularly microglia 69. This is attributed to the increase in endothelial vesicles, which enhances transcellular transport in the first few hours after stroke, while BBB disruption occurs 48 h post-stroke 62. Thus, the optimal liposome administration window is 48 h post-stroke, maximizing drug accumulation in parenchymal tissue and improving therapeutic efficacy for alleviating inflammatory brain injury. Additionally, Han *et al*. further discovered that lipids from different sources also affect nanoparticle biodistribution. Lipids from mouse brain tissue accumulated more in ischemic brain regions compared to those from liver tissue due to homologous interactions 63.

### 3.2. Polymeric nanoparticles

Polymeric nanoparticles represent a versatile class of nanocarriers that have garnered significant attention for IS therapy, owing to their tunable physicochemical properties, controllable drug release kinetics, and potential for functionalization. In contrast to liposomal systems, polymeric nanoparticles offer greater structural diversity and synthetic flexibility, enabling the rational design of carriers that are specifically tailored to navigate distinct biological barriers and meet therapeutic requirements 72-76. Polymers, including natural polymers (e.g., hydrogel and polysaccharides) and synthetic polymers (e.g., poly (lactic-co-glycolic acid) (PLGA) and polyamidoamine (PAMAM)), have been extensively utilized in the design of nano-DDS for IS (**Table 1**). This section highlights key categories of polymeric nanocarriers, such as PLGA, advanced polymeric micelles, and PAMAM. We aim to an in-depth, mechanism-driven summary of recent developments in polymeric nanomedicine, emphasizing their distinctive structural features, functional benefits, and their significance in current IS drug delivery studies.

PLGA, a linear copolymer of lactic and glycolic acids, stands as one of the most commonly employed biodegradable polymers in nanomedicine. Its well-defined degradation profile and FDA-approved status make it a benchmark material for delivering anti-ischemic stroke drugs, including antithrombotic agents, neuroprotective agents, and gene drugs 64-67. However, conventional PLGA nanoparticles suffer from rapid clearance by the reticuloendothelial system (RES), limiting their circulation half-life and target accumulation 68. To address this, structural modifications such as PEGylation to form PEG-PLGA copolymers have been employed. For example, Zamanlu *et al*. demonstrated that tPA conjugated to PEG-PLGA nanoparticles (tPA-PEG-PLGA NPs) exhibited prolonged circulation and enhanced thrombolytic activity compared to non-PEGylated nanoparticles, highlighting the role of surface hydrophilicity in modulating pharmacokinetics 67. Beyond stealth modifications, the incorporation of targeting ligands allows for the precise delivery of therapeutics to pathological sites. The use of thrombolytic drugs (e.g., tPA and uPA) and antiplatelet agents is often associated with dose-limiting systemic bleeding, a major adverse effect that narrows their therapeutic window. In this context, Zhang *et al.* functionalized PEG-PLGA nanoparticles with cyclic RGD (cRGD) peptides, enabling targeted interaction with GPIIb/IIIa receptors on activated platelets 74. This structural modification not only enhanced thrombus accumulation but also reduced off-target effects, emphasizing the critical role of surface ligand chemistry in enabling selective drug action.

Despite these advancements, traditional chemical conjugation methods often involve complex reactions and inefficient purification processes. To simplify functionalization, polydopamine (PDA), a versatile adhesive material, has been introduced as a surface coating. Park *et al.* demonstrated that PDA can be polymerized on PLGA nanoparticles under mild conditions, enabling the subsequent coupling of thiol- or amine-terminated ligands without the need for specific chemical handles 69. This strategy not only streamlines the modification process but also imparts intrinsic antioxidant properties, which help mitigate ROS-mediated injury in IS. For instance, Yin *et al.* and Xiao *et al.* employed PDA coatings to conjugate stroke-targeting peptides onto polymer nanoparticles, thereby enhancing both targeting efficiency and neuroprotection 70, 71.

**Figure 5:**
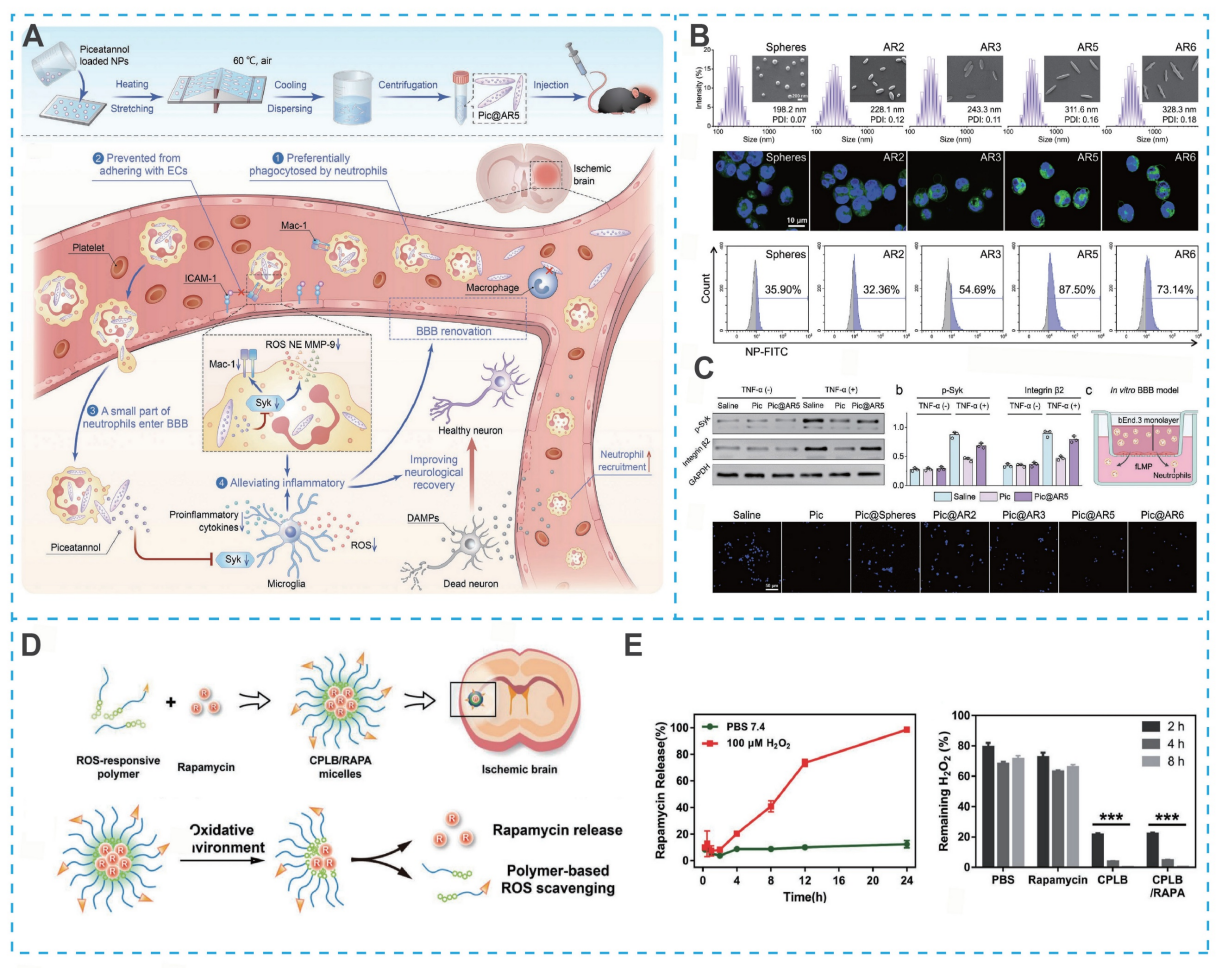
** Polymeric nanoparticle-based nano-DDS for IS treatment.** (A) Schematic of ischemic stroke therapy mediated by Pic@AR5 nanoparticles. (B) Effect of particle shape on the phagocytic efficiency of neutrophils. (C) Investigation of neutrophil intervention and inflammatory alleviation of activated microglia. Reproduced, with permission, from 73. Copyright (2023), Wiley-VCH GmbH. (D) Illustration of CPLB/RAPA micelle formation. (E) Illustration of micelle disassembly under oxidative environment and ROS responsiveness and scavenging ability of micelle formulations. Reproduced, with permission, from 49. Copyright (2019), Wiley-VCH GmbH.

Surface chemistry is widely recognized for its crucial role in influencing nanoparticle behavior, yet the physical characteristics, especially nanoparticle shape, also significantly impact biological interactions and therapeutic outcomes. Specifically, the shape of nanoparticles directly affects their delivery efficiency and therapeutic efficacy. Among the various factors, the aspect ratio (AR) of PLGA nanoparticles has been shown to substantially modulate their interaction with cells and tissues. For example, rod-shaped PLGA nanoparticles exhibit enhanced phagocytosis by neutrophils, which is particularly advantageous for targeting inflammatory sites 72. Song *et al.* developed a series of PLGA nanoparticles with variable AR (**Figure 5A**) 73. Among them, PLGA nanoparticles with an AR of 5 exhibited the highest neutrophil uptake and were subsequently loaded with piceatannol to form Pic@AR5 (**Figure 5B**). Benefiting from the efficient endocytosis of Pic@AR5 by neutrophils, piceatannol could significantly inhibit the inflammatory response (**Figure 5C**) 73.

Complementing their structural modifications, PLGA-based nano-DDS also exhibit multifunctional capabilities by integrating versatile therapeutic strategies. Photothermal therapies (PTT) have emerged as promising partners for thrombolytic drugs due to their synergistic mechanisms, which overcome the limitations of conventional thrombolysis. These modalities function by generating localized heat, contributing to efficient thrombus ablation. The encapsulation capacity of PLGA nanoparticles allows for the co-delivery of thrombolytic drugs with photothermal agents, creating a multifunctional system that amplifies therapeutic efficacy while circumventing systemic side effects 77, 78. For instance, Zhang *et al.* developed a multifunctional nanosystem by co-encapsulating uPA and the photothermal agent indocyanine green (ICG) into cRGD-functionalized PEG-PLGA nanoparticles (cRGD-ICG-uPA NPs) 74. The cRGD-ICG-uPA NPs exposed to light exhibited a significantly stronger thrombolytic effect (up to 72%) compared to free uPA or cRGD-ICG-uPA NPs without light exposure. This demonstrates the potential of combining thrombolytic drugs with PTT for enhanced thrombolytic efficacy 74.

Building upon the functional versatility of PLGA-based platforms, the growing focus has shifted toward the design of intelligent, microenvironment-responsive nanocarriers. Consequently, advanced polymeric micelles have emerged as a prominent class of materials for enhanced drug delivery. The complex pathological microenvironment of IS provides a diverse toolbox for the design of stimulus-responsive polymeric micelles. A representative strategy targets the acidic pH of the ischemic penumbra (pH ~6.5-6.8). Li *et al.* designed a polymer nanoparticle composed of amphiphilic polymer hyaluronic acid-poly (β-amino ester) (HB) for brain delivery of DNase 1. The poly(β-amino ester) component underwent protonation within the acidic microenvironment, prompting rapid disassembly of the nanoparticles and subsequent release of encapsulated DNase I. The released DNase I effectively inhibited platelet activation and neutrophil aggregation, thereby reducing microthrombosis formation and mitigating neuroinflammatory responses 74.

Furthermore, oxidation-responsive groups serve as a core design principle for developing novel polymeric micelles capable of reacting to the highly oxidative microenvironment in IS. Lu *et al.* developed a ROS-responsive micellar system (C-PEG-LysB) by integrating hydrophilic PEG with a hydrophobic polylysine core grafted with phenylboronate esters. The micelle surface was further functionalized with the fibronectin-binding peptide CREKA to confer active targeting capability (**Figure 5D**). Under the highly oxidative ischemic conditions, the phenylboronate esters underwent cleavage, leading to micellar disintegration and rapid release of the encapsulated mTOR inhibitor rapamycin (RAPA) (**Figure 5E**). Notably, this rational design not only enables controlled drug release but concurrently scavenges ROS, thereby conferring a dual therapeutic function that mitigates oxidative damage while enhancing pharmacological efficacy 49.

Beyond environmental factors such as pH and ROS, the pathological overexpression of specific enzymes, particularly thrombin, offers a distinctive mechanism for driving programmable structural transformations in nanocarriers. Guo *et al.* developed a size-shrinkable polymeric micelle (ASNPs) designed to respond to thrombin. The core structure of the micelle consists of PEG, poly-ε-caprolactone (PCL), and thrombin-cleavable peptide linkers. The initial targeting was achieved by surface-conjugated AMD3100, which binds to CXCR4 upregulated in the ischemic region. When the enzymatic cleavage peptides within ASNPs were ruptured by thrombins overexpressed in stroke sites, resulting in a substantial decrease in the hydrodynamic diameter of nanoparticles 50. This programmed size reduction was critical as it significantly enhances the diffusion and penetration of the nanoparticles into the ischemic brain parenchyma, leading to a 30-fold increase in drug accumulation compared to non-shrinkable, non-targeted controls.

Genetic therapeutics offer significant advantages over small-molecule or protein drugs in IS treatment, particularly due to their potential for long-lasting effects and the ability to target specific molecular pathways. This is especially important in stroke therapy, where gene drug-based strategies could address underlying pathophysiological processes like neuroinflammation and neuronal repair. However, hinges on the development of safe and efficient nanocarriers capable of overcoming the unique barriers to gene delivery. In this domain, dendrimers such as polyamidoamine (PAMAM) have emerged as particularly promising vectors, distinguished by several inherent advantages over preceding polymer nanoplatforms: (i) low immunogenicity; (ii) low host chromosome integration; (iii) low DNA recombination; and (iv) relatively high gene delivery efficiency. Additionally, it has been demonstrated that PAMAM dendrimers exhibit an innate homing capacity to sites of neuroinflammation. Taken together, Lee *et al*. engineered a PAMAM dendrimer covalently coupled with histidine and arginine (PG2HR) for the delivery of anti-inflammatory HO-1 plasmids. With enhanced cationic histidine- and arginine-mediated cellular uptake, endosomal escape and excellent serocompatibility, PG2HR had lower cytotoxicity and high gene drug delivery efficiency 75. Despite these promising characteristics, the clinical translation of dendrimers still requires further investigation into their long-term biodegradation and *in vivo* safety profiles 76.

### 3.3. Inorganic nanoparticles

While polymeric nanocarriers offer considerable versatility in drug delivery, their therapeutic efficacy in ischemic stroke can be limited by insufficient intrinsic bioactivity and constrained functionality in complex pathological environments. Inorganic nanomaterials including carbon nanotubes, fullerenes and mesoporous silicas have broadened the scope of applications in nano-DDS with their unique advantages: (i) high specific surface area providing more space and binding sites for drug loading and good physical and chemical stability; (ii) inherent spectral properties endow with real-time monitoring of drug delivery effects *in vitro and in vivo*; and (iii) the structural parameters allow for precise tuning and easier functional modifications of the surface 35, 77. For instance, to overcome the limitations of nerve growth factor (NGF) in neurological treatments, such as its short half-life and rapid degradation, Parichehr Hassanzadeh *et al.* exploited the high surface area and structural robustness of multi-walled carbon nanotubes (MWCNTs) to create stable MWCNT-NGF complexes. The MWCNT-NGF complexes demonstrated a significantly prolonged neuroprotective effect compared to free NGF, highlighting the ability of MWCNTs to not only stabilize therapeutic biomolecules but also enhance their efficacy over extended periods, providing versatile carriers for stabilizing and delivering biomolecules 78.

In addition to serving as passive carriers, several inorganic nanoparticles exhibit intrinsic therapeutic interventions, making them particularly suitable for addressing key pathological processes in IS 79, 80. Selenium, as one of the essential trace elements in the body, has been discovered to regulate redox homeostasis in the body with its inherent antioxidant activity as well as participation in the regulation of neurogenesis. Consequently, some selenium nanoparticles (Se NPs) have been exploited for IS treatment 81-83. To further enhance the *in vivo* brain targeting of Se NPs, Hamed Amani *et al.* synthesized for the first time PEGylated and OX26 antibody-functionalized Se NPs with a diameter of approximately 12 nm, which leverage transferrin receptor-mediated transcytosis to achieve enhanced accumulation in ischemic brain regions 81. Further mechanistic studies revealed that these Se NPs modulate multiple signaling pathways involved in regulation of cell metabolism, neuronal function, autophagy and apoptotic cell death as well as oxidative stress and inflammatory responses 81.

**Figure 6:**
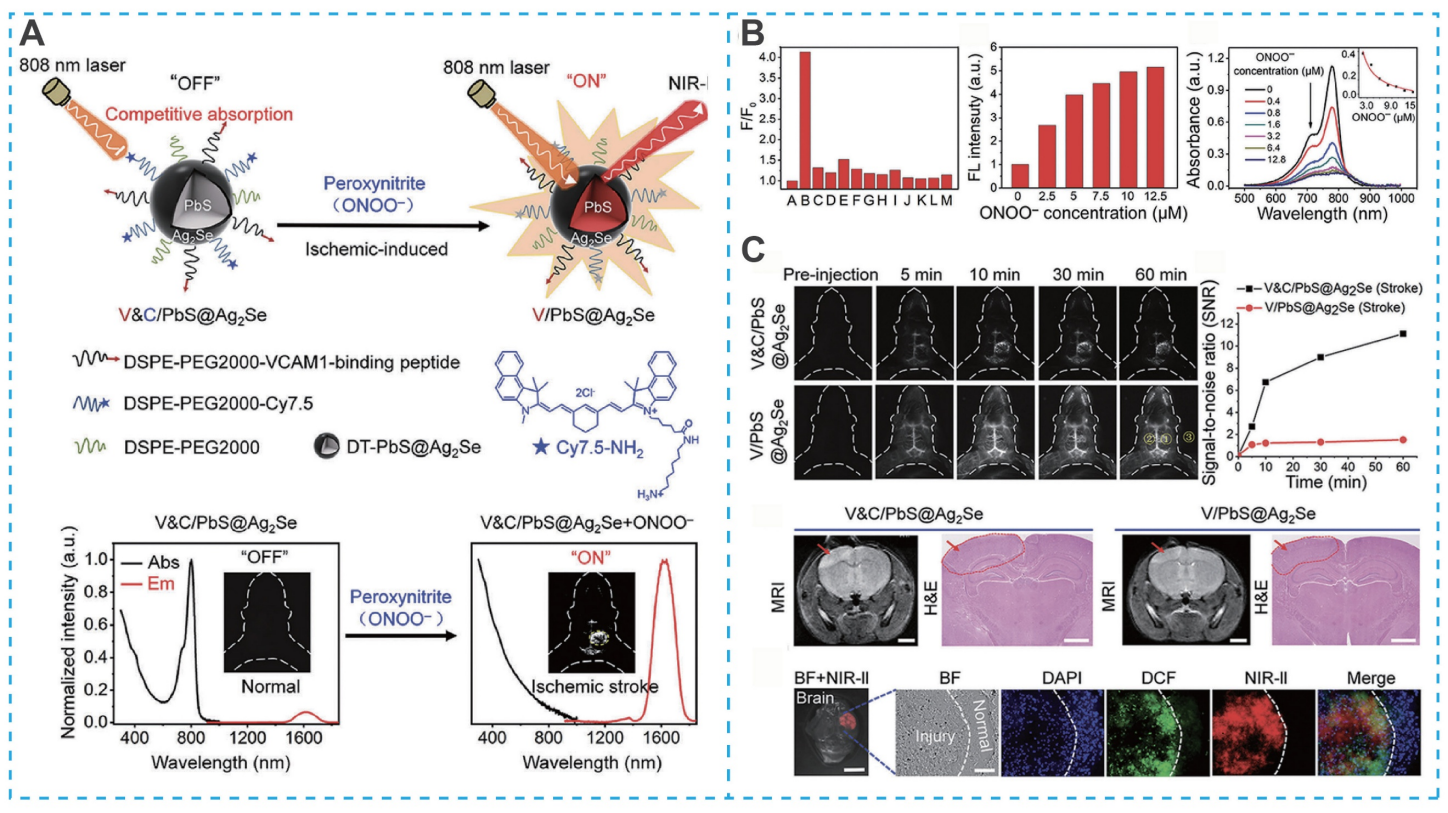
** Inorganic nanoparticle-based nano-DDS for IS treatment.** (A) Schematic illustration of the construction of the V&C/PbS@Ag_2_Se nanoprobe and detection of ONOO^-^ in ischemic stroke mice. (B) V&C/PbS@Ag_2_Se response to endogenous ONOO^-^ in HUVECs cell (A: Blank, B: ONOO^-^, C: H_2_O_2_, D:^1^O_2_, E: OH, F: Na^+^, J: K^+^, H: Ca^2+^, I: Mg^2+^, J: Zn^2+^, K: Cl^-^, L: HCO_3_^-^, M: HPO4^2-^). (C) V&C/PbS@Ag_2_Se for the detection of early ischemic stroke injury. Reproduced, with permission, from 84. Copyright (2021), Wiley-VCH GmbH.

Beyond their therapeutic functions, the unique optical and electronic properties of inorganic nanomaterials have enabled significant advances in real-time diagnostic imaging for IS. For example, Yang *et al.* designed a targeted activatable near-infrared IIb (NIR-IIb) nanoprobe (V&C/PbS@Ag₂Se) for highly sensitive detection of early cerebral ischemia 84. This nanoprobe employed a sophisticated "off-on" fluorescence activation strategy. Under initial conditions, fluorescence was quenched due to competitive absorption between Cy7.5 fluorophores and PbS@Ag₂Se quantum dots (**Figure 6A**). Upon intravenous administration, the system was directed to the inflamed vascular endothelium in ischemic regions via a VCAM1-binding peptide. Importantly, the probe underwent selective activation through the oxidation of Cy7.5 by peroxynitrite (ONOO⁻), a key biomarker of ischemic stroke (**Figure 6B and Figure 6C**) 84. This activation results in the immediate and highly specific fluorescence of ischemic lesion areas, enabling precise identification of early-stage ischemic events with high sensitivity.

Other metal oxide nanoparticles, such as cerium dioxide, have also been widely investigated for IS treatment due to their enzyme-mimetic antioxidant activity, which will be discussed in Section 3.5. Undeniably, inorganic nanoparticles possess unique properties, such as catalytic activity, imaging capabilities, and therapeutic functions, making them promising candidates for IS therapeutics. However, their clinical translation is hindered by several unresolved challenges, including long-term biosafety concerns, such as potential accumulation in peripheral organs, unpredictable biodegradation, and limited understanding of immune interactions 35, 77, 85.

### 3.4. Carrier-free self-assembly nanoassemblies

Although carrier materials-based nanodrug delivery systems are available for IS treatment, there are still some limitations in conventional nanocarriers, such as suboptimal stability, premature drug leakage, low drug loading capacity, and carrier excipients-relevant potential toxicity 104-106. In addition, the complex manufacturing process of some traditional nanocarriers is caused by low reproducibility and difficulties in large-scale production, resulting in multiple roadblocks to clinical translation. Large bodies of work have sought to improve nanocarriers to overcome the current deficiencies, but the outcomes were far from satisfactory 106.

Recently, some polymeric prodrugs or molecular drugs have been detected to self-assemble to form nanostructures by various interaction forces such as hydrophobic forces, hydrogen bonding forces, and π-π stacking forces, providing a potential avenue to develop efficient nanomedicines for IS treatment 107-112. It is widely believed that the low drug accumulation in the brain is a long-term difficulty in treating brain-related diseases. Notably, Molecularly self-assembled nano-DDS have high drug loading capacity due to drugs or prodrugs as carriers and cargos (**Table 2**), which will open the mind to design more potential nano-DDS for the treatment of IS. Prodrugs are inactive or less active compounds obtained after structural modification of the drugs* in vitro* and are transformed into active drugs and exert effects* in vivo*. Insertion of stimulus-responsive linkers in prodrugs endows the nano-DDS with capable of the specific-sites (thrombotic or brain injury sites) release of active drugs, thus significantly improving efficacy and reducing toxicity to normal tissues. In this section, we will discuss molecularly self-assembled nanodrugs, including polymeric prodrug-nanoassemblies, small-molecule drug-nanoassemblies and peptide/gene drug-nanoassemblies for treating IS.

#### 3.4.1. Polymeric prodrug nanoassemblies

Several polymers (e.g., polyethylene glycol and dextran) have been employed to construct polymeric prodrug-nanoassembly delivery systems for IS therapy 107, 108, 110, 111. These polymers are generally characterized by favorable biocompatibility, safety and non-toxicity as well as highly active chemical modification sites (e.g., hydroxyl, amino and carboxyl sites). Therefore, drugs as well as various thrombus-targeting or brain-targeting molecules could be covalently coupled to polymer chains through these chemical modification sites.

Among various polymers, dextran-related derivatives have been most commonly utilized to design the polymer-drug conjugates by virtue of inherent advantages such as excellent stability, biocompatibility and biodegradability as well as rich hydroxyl functional groups 113, 114. These hydroxyl sites provide an excellent platform for designing polymer prodrugs, effectively addressing adverse reactions such as the bleeding limitations associated with thrombolytic drugs. For instance, Liu *et al*. developed a targeted nano-DDS for uPA. In this system, uPA was conjugated to oxidized dextran (Oxd) through a pH-sensitive imine bond, enabling selective drug release in the acidic thrombus microenvironment (pH < 6.8) and minimizing off-target bleeding risks 110. The conjugate was further functionalized with an RGD peptide (uPA-Oxd-RGD), which targets the GPIIb/IIIa integrin on activated platelets, resulting in enhanced thrombolytic efficacy in a transient middle cerebral artery occlusion (tMCAO) model compared to free uPA. Notably, the uPA-Oxd-RGD system was also shown to attenuate BBB disruption by downregulating MMPs (MMP-2 and MMP-9), thereby potentially extending the time window for safe thrombolysis 110.

**Figure 7:**
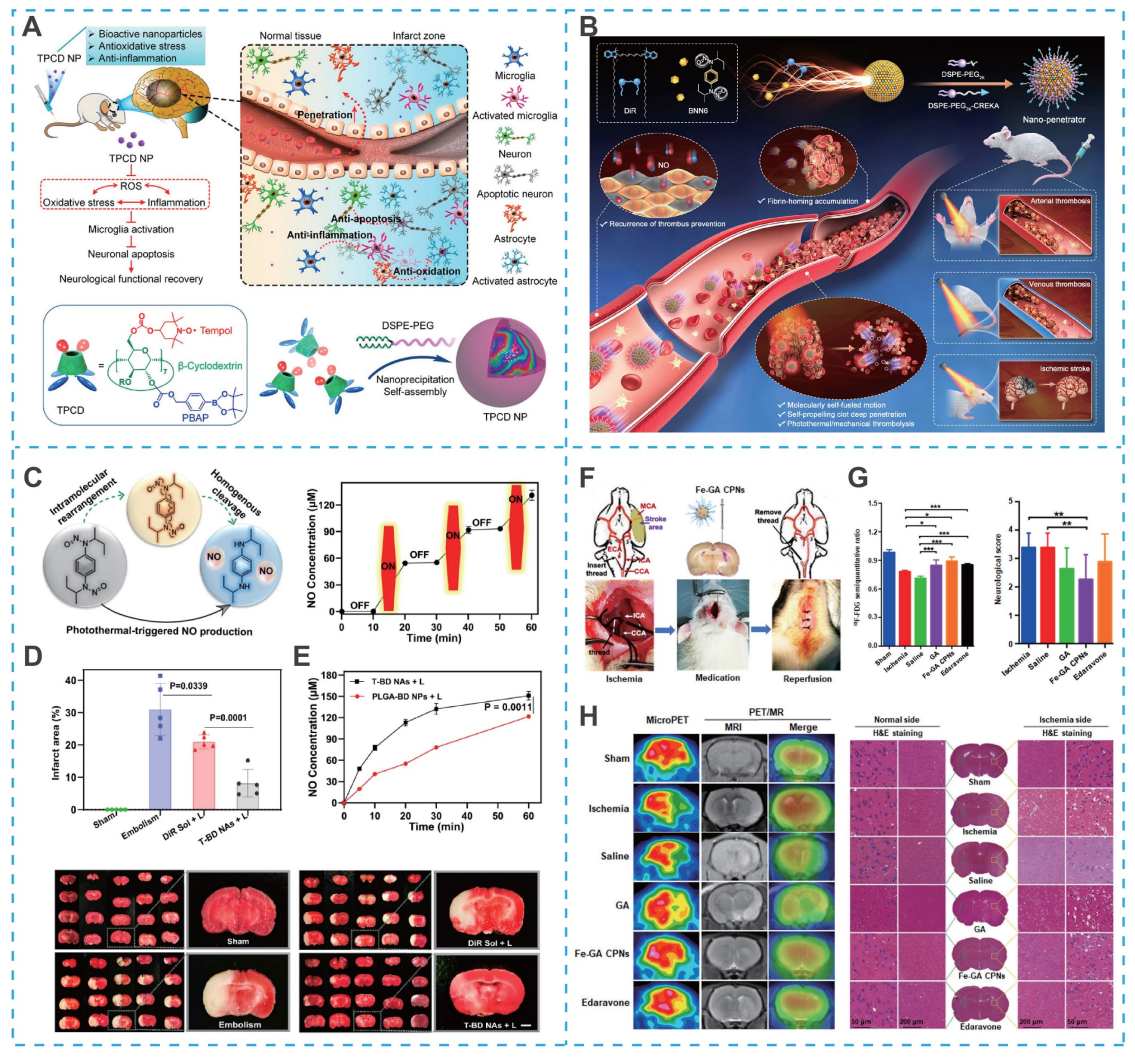
** Carrier-free self-assembly nanoassembly-based nano-DDS for IS treatment.** (A) Sketch showing targeted treatment of ischemic stroke by a multifunctional nanotherapy and the underlying mechanisms and engineering of the nanotherapy using a cyclodextrin-derived bioactive material TPCD. Reproduced, with permission, from 111. Copyright (2021), American Chemical Society. (B) Schematic of molecularly self-fueled nano-penetrator for nonpharmaceutical treatment of thrombotic diseases. (C) Chemical mechanisms of NO generation from BNN6 and off-on NO generation variations by the switch of laser irradiation. (D) Brain infarct volumes and representative photograph. (E) Laser-triggered NO release from T-BD NAs and PLGA-BD NAs. Reproduced, with permission, from 119. Copyright (2023), Nature. (F) Schematic illustration of model operation and injection workflow. (G) Results of the uptake ratio of the ischemic brain to the normal brain and the neurological function score. (H) MicroPET and PET/MR evaluation of rat brain after the intravenous injection of ^18^F-FDG after treatment and representative images of brain slices via H&E staining. Reproduced, with permission, from 120. Copyright (2023), Wiley.

Cyclodextrins, with their distinct structural characteristics, a hydrophilic outer surface and a hydrophobic central cavity, demonstrate exceptional molecular encapsulation and self-assembly abilities, surpassing those of linear polysaccharides like dextran 115, 116. These cyclic oligosaccharides, particularly β-cyclodextrin, which consists of seven glucopyranose units, are extensively utilized in nanomedicine. Their versatile host-guest interactions, chemical modifiability, and cost-effectiveness further contribute to their widespread application. Capitalizing on these properties, Yuan *et al*. utilized the hydroxyl active site on β-cyclodextrin to covalently graft the free radical-scavenging compound (Tempol) and ROS-responsive block phenylboronic acid pinacol ester (PBAP), which could be self-assembled to form stable nanoparticles (TPCD NPs) with the aid of lecithin and DSPE-PEG (**Figure 7A**) 111. Benefiting from the efficient and specific delivery of Tempol, TPCD NPs significantly reduced the infarct volume and accelerated the recovery of neurological function in mice with tMCAO 111.

#### 3.4.2. Small-molecule drug/prodrug nanoassemblies

Although polymeric prodrug-nanoassemblies are usually characterized by favorable colloidal stability and long circulation time *in vivo*, there are still some existing limitations: (i) Uncertainties in the synthesis of polymer-drug conjugates; (ii) relatively low drug-loading capacity; and (iii) potential polymer-related toxicity. In contrast, small-molecule drug/prodrug-nanoassemblies (SMDNPs) with distinct advantages, including ultrahigh drug loading and negligible excipient-induced adverse effects, have been extensively investigated 104. Moreover, their straightforward preparation further facilitates clinical translation and large-scale production in the future.

While SMDNPs offer attractive features, their formation strictly depends on specific intermolecular interactions (e.g., hydrophobic, π-π stacking, and hydrogen bonding forces), which inherently limits the range of therapeutics applicable for this strategy in IS. Fortunately, Zhang *et al.* identified several natural small-molecule antioxidants isolated from medicinal plants such as betulinic acid (BA), lupeol (LP), glycyrrhetic acid (GA), stigmasterol (ST) and oleanolic acid. They could spontaneously self-assemble into nanoparticles, with BA showing the most potent antioxidant activity 112. To further improve brain targeting and site-specific drug release, Zhang *et al.* further developed a pH-responsive self-assembled nanoparticle (A-BAM NP) based on betulinic amine (BAM), a derivative of BA, functionalized with AMD3100 for CXCR4-mediated ischemic homing 112. Additionally, A-BAM NP was co-loaded with the neuroprotective peptide NA1 and demonstrated triggered release of both agents under the acidic ischemic microenvironment, leading to synergistic neuroprotection and functional recovery *in vivo* 119. These works not only expand the library of self-assembling small molecules for IS treatment but also illustrate their dual role as both therapeutic agents and modular self-delivery carriers.

Self-assembling strategies have also been applied to phototheranostic agents. For instance, photosensitizer 1,1'-dioctadecyl-3,3,3',3'-tetramethylindotricarbocyanine iodide (DiR) can form self-assembled nanostructures for photothermal thrombolysis 117, 118. Notably, photothermal photosensitizers can be used for image-guided hyperthermic thrombolysis, effectively promoting the deep penetration of drugs into the thrombus. However, the insufficient efficiency of single photothermal thrombolysis and high thrombus recurrence rate limit the application of DiR. To overcome these drawbacks, Zhang *et al.* found that DiR and the NO donor N, N'-di-sec-butyl-N, N' dinitroso-1,4-phenylenediamine (BNN6) could be co-assembled into a self-fueled nano-penetrator. Nano-penetrator was further surfaced with DSPE-PEG_2K_ and DEPE-PEG_2K_-CREKA to improve colloidal stability and thrombus-targeting capability (**Figure 7B**) 119. Notably, the close molecular proximity between DiR and BNN6 within the co-assembled nanostructure enabled efficient energy transfer under laser irradiation, which triggered NO release via intramolecular rearrangement and homolytic cleavage of the N—NO bond in BNN6 (**Figure 7C**). The resulting bubbles propelled the nanomotor, enabling intelligent mechanical thrombolysis that acted synergistically with DiR-mediated photothermal therapy. Moreover, NO released from the nano-penetrator contributed to the prevention of thrombus recurrence and ameliorated cerebral injury in a MCAO rat model (**Figure 7D**). Importantly, as shown in **Figure 7E**, compared to PLGA nanoparticles loaded with equal proportions of DiR and BNN6, the carrier-free nano-penetrator exhibited more remarkable NO generation and self-motion due to the high fuel-loading capacity 119.

Beyond conventional therapeutic applications, self-assembled nanosystems have recently enabled integrated diagnostic and therapeutic strategies that are particularly valuable for managing the dynamic pathology of IS. In a notable example, ultrasmall iron-gallic acid coordination nanoassemblies (Fe-GA CPNs) were developed as antioxidant neuroprotectors with dual-mode PET/MR imaging capability for IS therapy (**Figure 7F**) 120. With their ultrasmall size and efficient ROS scavenging capacity, Fe-GA CPNs demonstrated significant cytoprotective effects in hydrogen peroxide-treated cells by restoring redox homeostasis (**Figure 7G**). PET/MR imaging-guided evaluation revealed distinct neurological recovery following Fe-GA CPNs treatment, confirmed by H&E staining in MCAO models (**Figure 7H**). Mechanistic studies indicated that Fe-GA CPNs inhibited neuronal apoptosis through restoration of Akt signaling while activating the Nrf2/HO-1 pathway. This dual-mode theranostic platform exemplifies how self-assembled nanosystems can simultaneously provide neuroprotection through multiple pathways while enabling real-time treatment monitoring.

Currently, although only a fraction of small-molecule drugs with self-assembly ability against IS have been discovered, considerable future work can be spent on exploring the self-assembly properties of other small-molecule drugs, and it is expected to expand the repertoire of SMDNPs with clinical potential for IS theranostics.

#### 3.4.3. Peptide/gene drug nanoassemblies

The field of molecular self-assembly has evolved from small molecules to encompass complex biomacromolecules, including therapeutic peptides and nucleic acids. In contrast to conventional small molecules, these biologics rely not only on basic intermolecular interactions but also on higher-order structural motifs (e.g., α-helices, β-sheets, and programmed base pairing) to direct their assembly into functional nanostructures. Biologics, including peptides, DNA, and RNA, have demonstrated considerable therapeutic potential in treating IS due to their high specificity and potent bioactivity. However, their clinical utility is often hampered by poor oral bioavailability, short plasma half-life, and rapid clearance by RES. While encapsulation within nanocarriers such as liposomes, dendrimers, or polymeric nanoparticles can improve stability and prolong circulation, challenges such as inefficient loading and complex manufacturing hinder clinical translation 64, 76, 121, 122. The emerging ability of certain peptides and nucleic acids to self-assemble into nanostructures offers a promising alternative for constructing efficient, carrier-free delivery systems 123-126.

Most peptide- or gene-based nanoassemblies developed for IS aim to modulate the pathological microenvironment of the brain and promote neurological recovery. For example, Yu *et al.* designed activatable protein nanoparticles (APNPs) formed by the self-assembly of three independent peptides, including NR2B9c, a promising type II neuroprotective peptide conjugated via two coiled-coil forming motifs jointed with a "self " peptide 126. Among them, the "self" peptide prolonged the circulation time *in vivo* of APNPs by inhibiting the clearance of macrophages in RES. Importantly, enzymatic reaction sequences (e.g., thrombin) could be designed between the therapeutic peptide (NR2B9c) and the coiled-coil motifs, providing options for designing enzyme-responsive nano-DDS to realize on-demand drug release. Furthermore, to further enhance the brain-targeting of APNPs, Wu *et al.* covalently modified CAQK or CCAQK (a selective homing peptide targeting the brain injury site) in APNPs by a click-condensation reaction with 2-chlorobenzolthiazole (CBT) 123. The results showed that CCAQK-conjugated APNPs exhibited distinct superiority over APNPs, such as longer half-life, greater targeting ability and higher injured brain-penetrated efficiency and stronger therapeutic efficacy.

In comparison to peptide drugs, the self-assembly processes of gene drugs are more challenging. Small interfering RNAs (siRNAs) are powerful tools to suppress gene expression and can be targeted to treat different diseases 127. However, siRNAs generated in polymerized form using DNA/RNA provided by nature are unable to self-assemble into nanoparticles 128. To address this conundrum, Lee *et al.* reported a self-assembled siRNA microsponge by employing an artificial long-stranded RNA transcript that incorporated a T7 promoter and a target sequence. Subsequently, micronsponge was converted into desired siRNAs under the action of the Dicer enzyme *in vivo* 128. In addition, the DNA circular template-based gene transcription technique is another artificial synthesis method to obtain spherical self-assemblies. Antisense oligonucleotides (ASO) are considered promising siRNAs by silencing the caspase-3 gene to exert anti-apoptotic effects for IS treatment 124. Yu *et al.* obtained a self-assembled spherical nucleic acid nanostructure (TD) using a circular DNA template encoding both caspase-3-targeting antisense oligonucleotides (ASOs) and a transferrin receptor (TfR)-targeting inducer. The TD enhanced BBB penetration by 6.4-fold through TfR-mediated transcytosis and, upon endo/lysosomal degradation, released ASOs that effectively suppressed caspase-3 expression, reducing apoptosis and exerting neuroprotective effects 124.

Although peptide/gene nanoassemblies offer high biological specificity and potent therapeutic activity for directly targeting key pathological pathways in IS, this advanced functionality entails greater complexity. For instance, the self-assembly of biomacromolecules often requires more precise control over intermolecular interactions. Additionally, there are more substantial challenges in stability, biosafety, and large-scale reproducibility compared to polymeric prodrug and small-molecule nanoassemblies. Given these challenges, future research should focus on developing novel biomimetic self-assembly strategies, rationally designing modular peptide sequences and genetic components to achieve safe, precise, and adaptable therapeutic approaches for IS.

### 3.5. Nanozymes

Self-assembled nanomedicines typically rely on molecular/supramolecular interactions. However, an alternative approach has emerged that utilizes inorganic nanomaterials with intrinsic enzyme-mimetic activities, known as nanozymes. Oxidative stress-mediated brain tissue injury plays a central role in IS pathology 129. Moreover, the ischemic region continues to produce excessive RONS following reperfusion by thrombolytic therapy, exacerbating neurological damage 15. Although natural antioxidant enzymes normally scavenge RONS, their expression is inhibited during disease onset, and they are insufficient to eliminate the excess RONS 129, 130. Early attempts to deliver exogenous natural antioxidant enzymes to the ischemic site yielded suboptimal outcomes 131. The limited clinical efficacy of these natural antioxidant enzymes may be attributed to the following drawbacks: (i) poor stability *in vitro* and inability to be stored or recycled for long-term use; (ii) rapid clearance *in vivo*; (iii) difficulty in penetrating the BBB; and (iv) complex isolation and purification processes, coupled with high production costs. Nanozymes offer a promising alternative by mimicking natural enzyme activities while overcoming these limitations. To date, a variety of nanozymes with antioxidant properties have been extensively investigated for IS therapy (**Table 2**).

**Figure 8:**
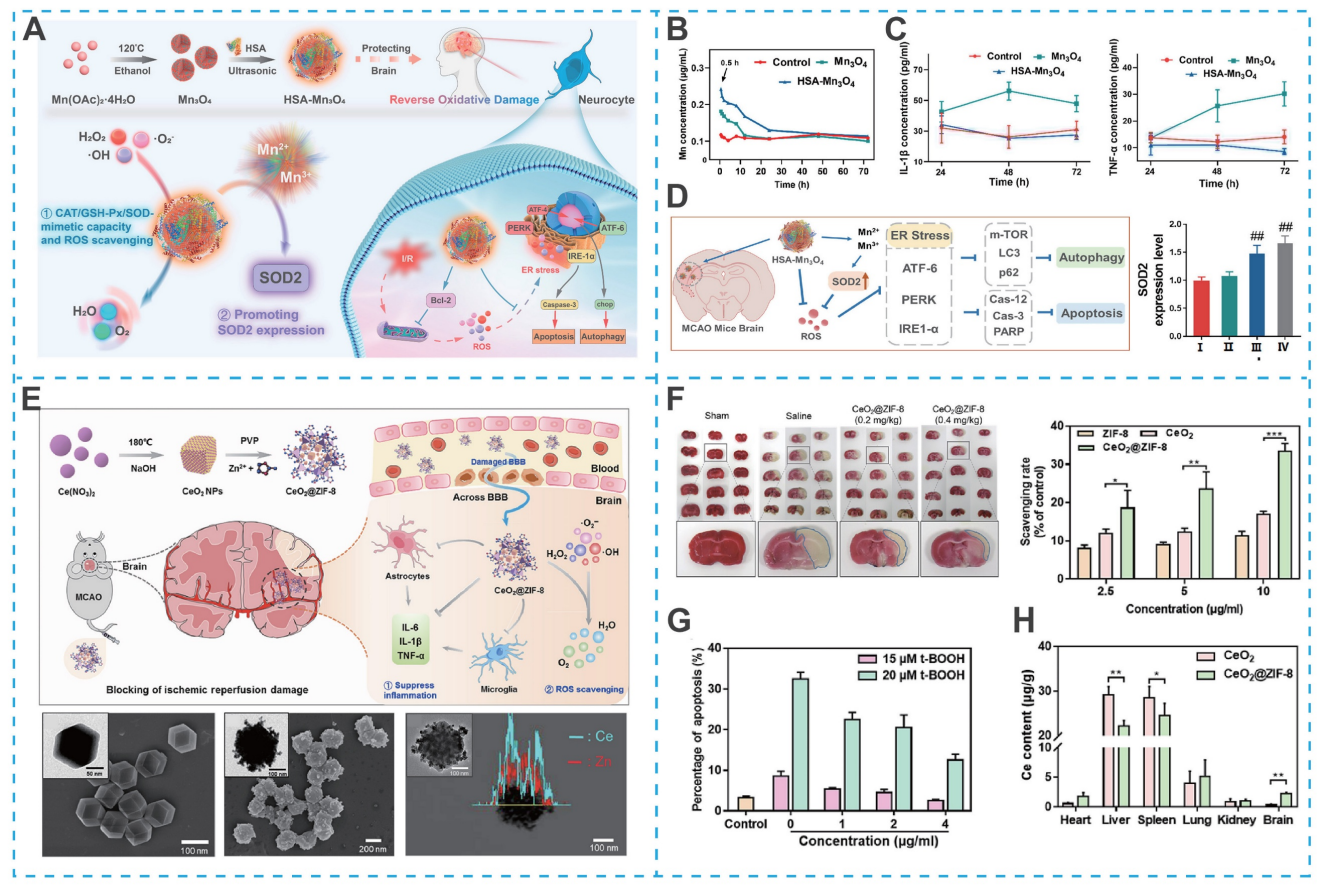
**Nanozyme**-**based nano-DDS for IS treatment.** (A) Schematic illustration of the HSA-Mn_3_O_4_ synthetic route and ROS scavenging mimetic-enzyme capacity in reperfusion-induced injury in ischemic stroke. (B) ICP-MS detection of Mn content in serum after intravenous injection with Mn_3_O_4_ and HSA-Mn_3_O_4_. (C) Enzyme-mimetic ROS scavenging capacity of HSA-Mn_3_O_4_. (D) Effect of HSA-Mn_3_O_4_ on hypoxia-, apoptosis-, autophagy- and ER stress-related pathways in the brain tissue of MCAO mice. Reproduced, with permission, from 133. Copyright (2022), American Chemical Society; (E) Schematic illustration for in situ synthetic approach of CeO_2_@ZIF-8 nanotherapeutics (F) CeO_2_@ZIF-8 reduces infarct volume by reducing ROS-induced oxidative damage. (G) CeO_2_@ZIF-8 reduces t-BOOH-induced PC12 cell apoptosis. (H) Biodistribution of Ce in the mice main organs after intravenous injection with CeO_2_ and CeO_2_@ZIF-8. Reproduced, with permission, from 134. Copyright (2020), Science.

Despite the promising potential of nanozymes in blocking overproduction or removing RONS, there are still some drawbacks limiting their application, such as insufficient antioxidant activity and lack of stability *in vivo*. Recent efforts have focused on enhancing their catalytic performance and biosafety through rational material design. For instance, Liu *et al*. developed Co-doped Fe_3_O_4_ nanozymes with the ability to scavenge RONS for ameliorating ischemia/reperfusion-induced injury 132. Remarkably, the activity of the Co-doped Fe_3_O_4_ nanozymes was 100-fold higher than the Fe_3_O_4_ nanozymes alone, providing a guarantee for achieving excellent therapeutic effects. In another research, Huang *et al*. synthesized human serum albumin (HSA)-stabilized Mn_3_O_4_ nanozymes (HSA-Mn_3_O_4_), which exhibited enhanced stability, longer blood circulation time, and superior RONS-scavenging ability compared to bare Mn_3_O_4_ nanozymes (**Figures 8A-C**) 133. Significantly, HSA-Mn_3_O_4_ could inhibit hypoxia-induced apoptosis and endoplasmic reticulum stress, offering significant neuroprotective effects at ischemic sites (**Figure 8D**).

Notwithstanding these advances, nanozymes incorporating metal ions, while exhibiting potent antioxidant activity, may raise potential neurotoxicity concerns. For example, cerium dioxide (CeO₂) nanozymes, which mimic superoxide dismutase activity, hold promise for antioxidant therapy but face challenges related to biosafety and uncontrolled particle size. To address these issues, He *et al.* designed bioactive zeolitic imidazolium framework-8 capped CeO_2_ nanoparticles (CeO_2_@ZIF-8 NPs), effectively inhibiting lipid peroxidation in brain tissue, thereby reducing ischemia-induced neuron oxidative damage and apoptosis in tMCAO mouse models (**Figures 8E-G**) 134. Admittedly, the ZIF-8 surface-capped modification improved the safety, antioxidant activity, stability, and BBB permeability of CeO_2_ NPs, as well as achieving controllability of particle size (**Figure 8H**).

Prussian blue nanoparticles (PBNPs) have also gained attention as biocompatible nanozymes capable of scavenging ROS, providing a platform for the development of safe nanozymes. Since the specific surface area of PBNPs critically influences their RONS elimination efficiency, hollow-structured Prussian blue nanozymes (HPBZs) have been actively explored to maximize catalytic activity. However, the complex synthesis of such architectures has hindered their clinical potential 135. Recently, Zhang *et al*. reported a Bi³⁺-assisted, template-free strategy to fabricate HPBZs with multi-enzyme activities, providing a simple clinical-scale production of HPBZs and effectively protecting neurons from hypoxia- and ischemia-induced damage both *in vitro* and *in vivo* 136. Faithfully, an ideal nanozyme should integrate high catalytic activity, long-term *in vivo* safety and stability, prolonged circulation half-life, and straightforward synthesis.

## 4. The evolution of nanodrugs: toward bioinspired nanosystems

Conventional nano-DDS have significantly advanced IS therapy by improving drug stability, prolonging systemic circulation, and promoting penetration across the BBB. These advantages have laid an important foundation for nanoscale interventions. However, their clinical performance remains limited by several persistent challenges, including insufficient targeting accuracy, nonspecific biodistribution, rapid clearance by RES and especially the risk of immunogenicity. To transcend these barriers, research has pivoted toward novel biomimetic nanomedicines that exploit natural biological components to create more sophisticated and biologically integrated delivery systems.

### 4.1. Cytomembrane-camouflaged biomimetic nanomedicines

Among emerging strategies, cytomembrane-camouflaged nanomedicines have gained particular attention. By enveloping synthetic nanoparticles with natural cell membranes, these nanomedicines can effectively evade the immune system while retaining the surface proteins and biological functions of the source cells. This biomimetic design not only extends systemic circulation and enhances biocompatibility but also enables active targeting to thrombotic or inflammatory sites via intrinsic homing ligands. In this section, we systematically review the latest developments in erythrocyte membrane-camouflaged nanomedicines, platelet membrane-camouflaged nanomedicines, immunocyte membrane-camouflaged nanomedicines and other cytomembrane-based biomimetic nanomedicines and are summarized in **Table 3**, highlighting their unique advantages in achieving targeted drug delivery and modulating pathological processes in IS.

#### 4.1.1. Erythrocyte membrane-camouflaged nanomedicines

Erythrocytes, with their natural biocompatibility, biodegradability, and non-immunogenic properties, are widely recognized as ideal vehicles for long-circulation drug delivery. Their remarkable stealth properties are largely attributed to CD47, a "self-marker" protein abundantly expressed on the erythrocyte surface. CD47 binds to signal regulatory protein α (SIRPα) on phagocytic cells of the RES, including macrophages, delivering a potent "don't-eat-me" signal that reduces phagocytic clearance 163. For instance, Lv *et al*. developed a bioengineered nanocarrier, consisting of a dextran polymer core modified with ROS-responsive boronic ester and an erythrocyte membrane shell conjugated with stroke homing peptide (SHp) for the delivery of neuroprotective drug NR2B9C to ischemic brain 47. Subsequently, high levels of ROS in ischemic neurons triggered intracellular NR2B9C release, significantly improved the specific protective effect on the injured neurons. Notably, the modified erythrocyte membrane played an excellent role in prolonging the systemic circulation time of NR2B9C and enhancing active targeting, enabling an "intelligent drug release and camouflage" nanoplatform for precise IS therapy.

#### 4.1.2. Platelet membrane-camouflaged nanomedicines

**Figure 9:**
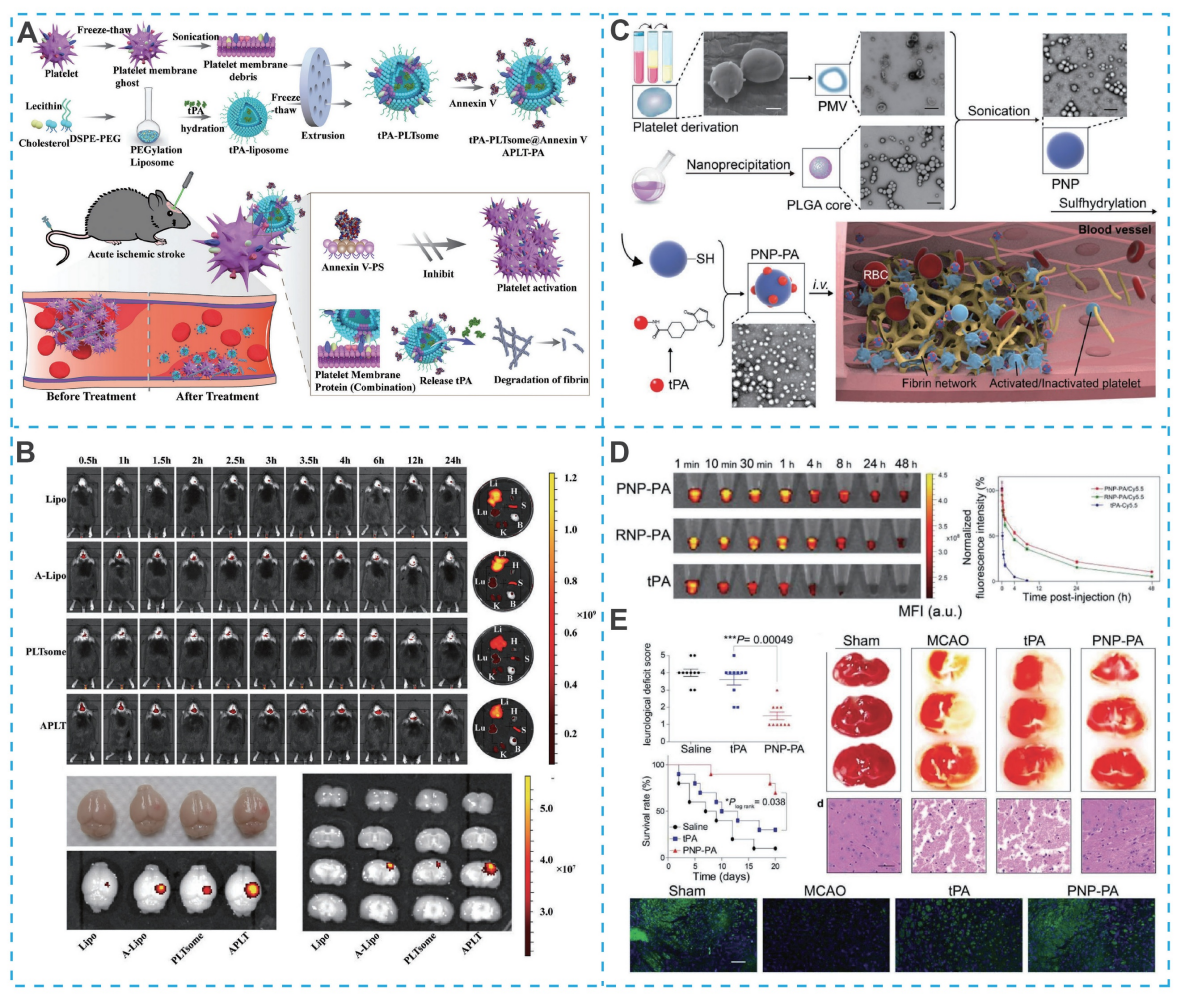
** Cytomembrane-camouflaged nano-DDS for IS treatment.** (A) Schematic of APLT-PA fabrication and its targeted thrombolysis for acute thromboembolic stroke. (B) The targeting efficacy* in vivo* of APLT-PA. Reproduced, with permission, from 165. Copyright (2022), Wiley. (C) Schematic illustration of the synthesis of PNP-PA nanoparticles. (D) Plasma fuorescence in blood from healthy mice treated with PNP-PA, RNP-PA, or rt-PA at the indicated time points. (E) Therapeutic effects of PNP-PA in the ischemic stroke mouse model. Reproduced, with permission, from 166. Copyright (2020), Wiley.

The natural biocompatibility of platelet membranes helps reduce non-specific clearance by the RES, but their immune evasion capability is weaker than that of erythrocyte membranes, due to the absence of the CD47 protein on the platelet membrane surface. Nevertheless, platelet membranes are rich in specific glycoproteins (such as GPIb-IX-V, GPIIb/IIIa, GPIa/IIa, and P-selectin), which preferentially adhere to damaged vascular endothelium and thrombus sites in IS lesions, providing another potential strategy for the design of novel nano-DDS for IS treatment 164. For example, Quan *et al.* designed a novel bionanomedicine (APLT-PA) to improve the efficiency of tPA for targeted thrombolysis, in which APLT-PA was composed of tPA-loaded liposomes embedded with annexin V and enveloped by platelet membrane fragments (**Figure 9A**) 165. Due to the thrombus-homing nature of platelet membranes and the inhibitory effect of membrane-linked protein V on activated platelets (via binding to exposed phosphatidylserine at thrombus sites), APLT-PA exhibited excellent targeting efficiency (**Figure 9B**), substantial thrombus lysis and significant improvement in neurological function in mice with acute IS. In addition to encapsulating drugs inside platelet membrane-camouflaged nanoparticles, drugs can also be directly attached to the platelet membrane surface. Xu *et al*. coated platelet membranes on the surface of PLGA polymers and chemically bound the thrombolytic drug tPA to engineered platelets (PNP-PA) (**Figure 9C**) 166.

Compared to free tPA, PNP-PA had a superior *in vivo* circulation time (**Figure 9D**).* In vivo* experiments showed a 3.5-fold increase in survival rate (up to 70%) in PNP-PA-treated MCAO mice (**Figure 9E**).

However, prolonged tPA use can cause bleeding as a side effect. Furthermore, tPA-mediated thrombolysis leads to a sudden increase in oxygen in the brain, generating ROS, which can cause reperfusion oxidative damage. Therefore, designing a bionic nano-DDS with efficient, site-specific thrombolytic effects and long-lasting neuroprotective effects is critical. Yu *et al*. developed a platelet-membrane-biomimetic nanovesicle co-loaded with melanin nanoparticles (MNP) and tPA for the sequential treatment of ischemic stroke. This platform enhances thrombus targeting through its biomimetic coating, enables precise NIR-triggered tPA release via melanin's photothermal effect at the thrombus site, and subsequently delivers small MNPs (~4.5 nm) that can cross the BBB to mitigate ischemia-reperfusion injury by scavenging free radicals and suppressing inflammatory and immune responses 167. In addition to external conditions precisely controlling the release of thrombolytic drugs, specific response release based on the thrombotic microenvironment (highly expressed thrombin) can also improve the bleeding side effects of tPA. Xu *et al*. designed a bioengineered "nanoplatelet" (tP-NP-tPA/ZL006e), which consisted of a dextran-derived polymer nanoparticle core loaded with the neuroprotective agent ZL006e and a platelet membrane shell coupled with a Tat peptide linked to thrombolytic agent tPA via a thrombin-responsive bond 168. Upon recruitment to the thrombus site, tPA was released by upregulated thrombin, and the Tat peptide enabled penetration of the blood-brain barrier to deliver ZL006e for site-specific neuronal protection, thus achieving the dual therapeutic effect of thrombolysis and neuronal protection at the ischemic site. Notably, although the natural thrombus homing properties of platelet membrane-camouflaged nanodrugs play an important role in thrombolytic therapy, they play a negligible role in the damaged brain parenchyma and still need to be complemented by other therapeutic tools.

#### 4.1.3. Immunocyte membrane-camouflaged nanomedicines

Nanoparticles camouflaged with erythrocyte and platelet membranes alone are not sufficient to independently reach the site of cerebral ischemia. However, it has been shown that various immune cells involved in the pathological processes of IS offer promising candidate cell membranes for creating bionic carriers with active targeting properties toward the damaged brain. Functionalized nanoparticles, such as those with monocyte membranes and rapamycin, have demonstrated synergistic chemoimmunotherapy effects in mitigating reperfusion-induced injury in IS 169, 170. The onset of IS triggers inflammatory responses that prompt brain microvascular endothelial cells to release recruitment signals to surrounding immune cells, including intercellular adhesion molecule-1 (ICAM-1) and P-selectin 171. Among these cells, neutrophils can strongly interact with brain microvascular endothelial cells due to their membrane expression of integrin β2, macrophage-1 antigen (Mac-1) and lymphocyte function-associated antigen 1 (LFA-1), making them promising brain-targeted bionanocytes, providing a high-quality platform for drugs that are trapped by low brain delivery efficiency 172. For example, the aforementioned nanozymes, despite their potent anti-inflammatory and anti-oxidative stress capabilities, are also not efficiently delivered to the damaged brain sites to exert their drug effects. To address this challenge, Feng *et al*. cleverly designed a neutrophil-like cell membrane-encapsulated mesoporous Prussian blue nanocyte enzyme (MPBzyme@NCM) to achieve active targeting of IS by exploiting the property that neutrophils tend to interact with brain microvascular endothelial cells at the site of inflammation 173. In a tMCAO mouse model, MPBzyme@NCM showed superior brain accumulation compared to naked MPBzyme, which in turn significantly enhanced the anti-inflammatory and antioxidant effects of MPBzyme at brain lesions. Interestingly, MPBzyme@NCM produced long-term therapeutic effects (28 days) by inducing microglia polarization toward the M2 phenotype, reducing neuronal apoptosis, and promoting the proliferation of neural stem and precursor cells, ultimately improving neurological function.

**Figure 10:**
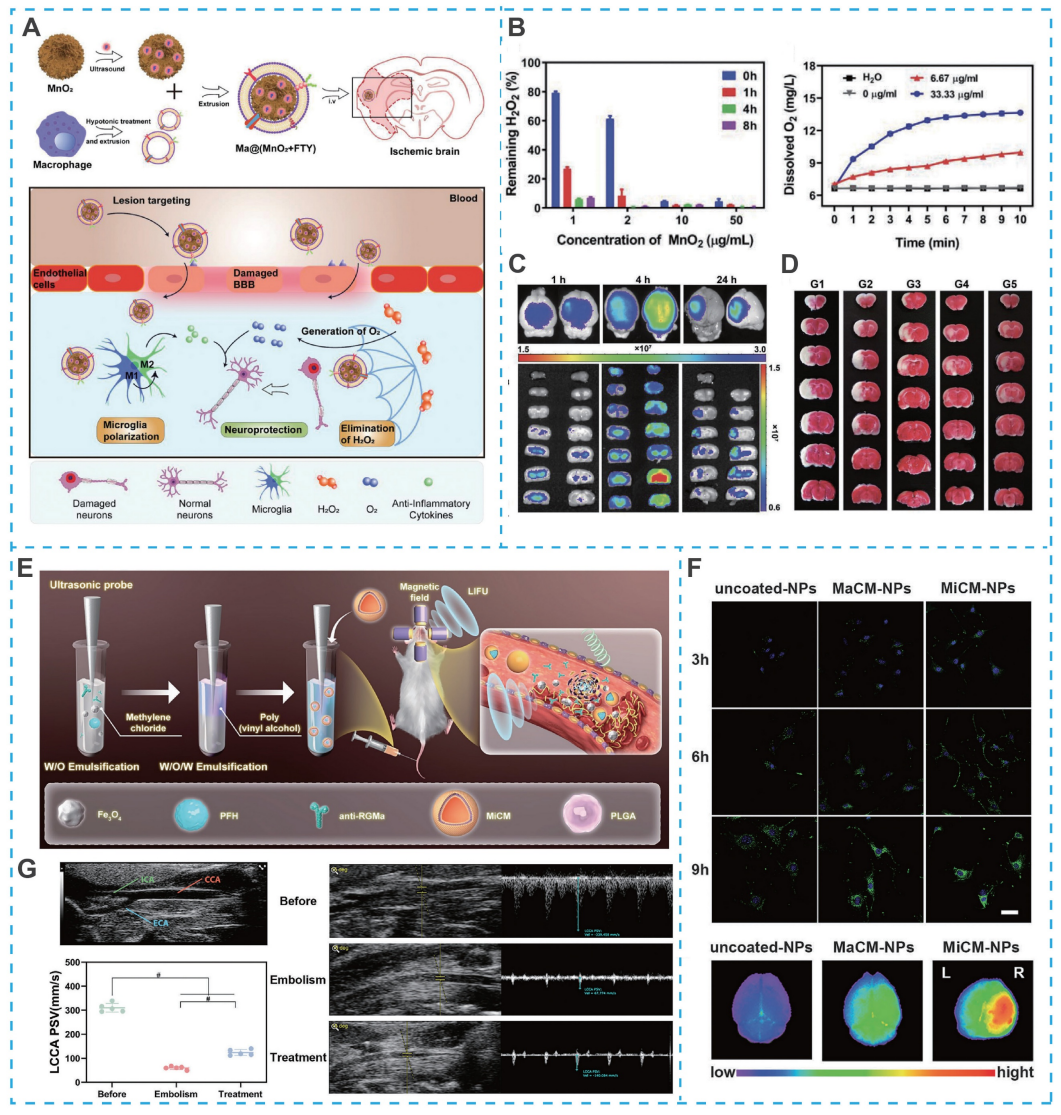
** Cytomembrane-camouflaged nano-DDS for IS treatment.** (A) Illustration of Ma@(MnO_2_+FTY) nanoparticles formation and salvation of damaged neurons in ischemic brain. (B) H_2_O_2_ scavenging behavior of MnO_2_ nanospheres and the changes of O_2_ concentration in H_2_O_2_ after MnO_2_ with different concentrations were added. (C) The targeting results of Ma@(MnO_2_+FTY) nanoparticles to the ischemic brain. (D) The rescue ability of nanoparticles on ischemic penumbra, brain sections were stained with TTC. Reproduced, with permission, from 170. Copyright (2021), Wiley. (E) Illustration of MiCM-NPs preparation. (F) Targeting ability of MiCM-NPs *in vivo* and *in vitro*. (G) B-mode and CEUS images of left common carotid artery embolization model of SD rats received uncoated-NP injection pre-LIFU irradiation and post-irradiation. Reproduced, with permission, from 175. Copyright (2024), Wiley-VCH GmbH.

Similarly, macrophages can not only enter the BBB by changing their shape in an exudative manner, but also their membrane proteins can recognize ICAM-1 on brain microvascular endothelial cells and pinpoint brain lesion areas. For example, Li *et al*. developed a macrophage-camouflaged foveolar manganese dioxide (MnO_2_) nanosphere loaded with fingolimod (FTY) (**Figure 10A**). In particular, elimination of overproduced ROS during ischemia effectively suppresse oxidative stress, but the insufficient oxygen microenvironment remains a stumbling block to neuronal recovery 170. Although the oxygen concentration increases rapidly after reperfusion, it also induces the regeneration of ROS, so the balance between ROS depletion and O_2_ delivery remains a high challenge. However, MnO_2_ nanoparticles could consume excess hydrogen peroxide (H_2_O_2_) and convert it to O_2_ in situ, successfully breaking the imbalance between ROS depletion and O_2_ delivery (**Figure 10B**). More importantly, due to the camouflage of macrophage membranes, MnO_2_ nanoparticles and FTY specifically targeted ischemic conditions and reduce oxidative stress and reversed the pro-inflammatory microenvironment by inhibiting multiple signaling pathways (e.g., NF-κB signaling pathway and STAT3 pathway), ultimately achieving a synergistic neuroprotective effect, overcoming the shortcomings of single-target therapy in IS treatment (**Figure 10C and Figure 10D**).

In contrast to the circulating neutrophils and macrophages discussed above, microglia, as the resident immune cells of the central nervous system, play a more direct and central role in regulating neuroinflammation following cerebral ischemia. Microglia can polarize into either pro-inflammatory M1 phenotypes or anti-inflammatory M2 phenotypes. Since M1 microglia promote neuroinflammation and neuronal dysfunction, shifting them to the M2 phenotype is a key strategy for treating IS. M2 microglia membranes not only possess the ability to cross the BBB and home to cerebral ischemia but also secrete anti-inflammatory cytokines and repolarize M1 microglia into the M2 phenotype. Duan *et al.* proposed a M2 microglia membranes-coated nano-scavenger consisting of self-assembled polyphenol tannic acid (TA) and catalase (CAT) (TPC NPs). Due to the BBB crossing and ischaemic-homing ability of M2 microglia membranes, TPC NPs specifically localized to ischemia, followed by the on-demand release of TA and CAT, leading to synergistic relief of inflammation and protection of neurons through multiple therapeutic mechanisms, including (i) TA and CAT exert potent free radical scavenging capacity; and (ii) M2 microglia membranes secrete anti-inflammatory cytokines and repolarise M1-phenotype microglia to M2-phenotype 174.

Building on the therapeutic potential of microglia membrane-coated systems, recent advances have further integrated real-time imaging capabilities into such platforms, enabling precise visualization of targeting and treatment processes. For example, Cheng *et al.* developed an artificial nanoplatform coated with microglia membrane (MiCM) and loaded with anti-repulsive guidance molecule, a monoclonal antibody (anti-RGMa) and superparamagnetic iron oxide (Fe₃O₄) for multimodal IS treatment (**Figure 10E**) 175. Following tail vein injection, the MiCM coating facilitated active targeting to ischemia-damaged endothelial cells (**Figure 10F**). Upon exposure to low-intensity focused ultrasound (LIFU), the platform released anti-RGMa and Fe₃O₄, inducing a "liquid-to-gas" phase transition of perfluorohexane (PFH) and magnetic field-driven movements to disrupt the thrombus, while anti-RGMa provided neuroprotection against ischemia/reperfusion injury (**Figure 10G**) 175. Notably, this nanosystem incorporated ultrasound/photoacoustic imaging functions, allowing non-invasive visualization of thrombus localization and treatment progress, particularly when thrombi were situated in extracranial arteries. This integrated imaging capability not only confirmed targeted accumulation but also enabled real-time monitoring of therapeutic efficacy, addressing a key limitation of conventional nanotherapeutics that lack feedback mechanisms.

#### 4.1.4. Other cytomembrane-based biomimetic nanomedicines

Although biomimetic nanocarriers based on erythrocyte, platelet, or neutrophil membranes offer favorable RES evasion and targeting capabilities, their limited availability poses challenges for large-scale clinical use. Recent studies have explored engineering cells available in larger numbers to create bionanoparticles with brain-targeting properties for IS treatment 176-178. For example, Shi *et al.* engineered mesenchymal stem cell (MSC) membranes overexpressing CXCR4 receptors, which specifically target inflammatory regions in the ischemic brain. Specifically, they stimulated MSC by iron oxide (Fe_3_O_4_) nanoparticles (20 nm) to obtain CXCR4 receptor-overexpressing MSC membranes and coated with A151-loaded polydopamine nanospheres (PDA), resulting in engineered CXCL12 bionic decoy integrated multifunctional immunosuppressive nanoparticles (VIN) 176. Among them, the oxidation of PDA by high ROS at the site of cerebral ischemia contributed to the loss of Zn^2+^ complexation, followed by the massive release of A151 loaded therein. A151 (oligonucleotide with telomerase repeat, 5'-TTAGGGTTAGGTTAGGGTTAGGG-3') inhibits the cyclic GMP-AMP synthase-stimulator of interferon genes (cGAS-STING) in microglia, leading to their polarization into anti-inflammatory M2 phenotype microglia to suppress the inflammatory response and promote the recovery of neurological function. At the same time, PDA not only responded specifically to the ischemic microenvironment for precise drug release, but also effectively scavenged the over-expressed ROS at the ischemic site, thereby dynamically balancing the redox microenvironment at the damaged site. Notably, the CXCR4-rich MSC membranes can act as decoys, preventing peripheral inflammatory cells from infiltrating the brain, thus suppressing inflammation from both activated M1 microglia and infiltrating immune cells.

Additionally, cancer cell membrane-mimetic nanocarriers offer another strategy for targeting IS. Since some malignant tumors develop brain metastasis in later stages, cell membranes can be used to design bionanoparticles with enhanced BBB penetration and targeting properties for IS treatment. Due to the high reproductive capacity of cancer cells, they are also ideal candidates for the large-scale production of membrane-coated nanoparticles. For instance, He *et al*. found that 4T1 cells can penetrate the BBB and specifically adhere to sites of brain inflammation in the presence of brain metastasis through two pathways: (i) syndecan-1 (CD138) on the cell membrane can specifically interact with brain vascular endothelial cells, platelets that preferentially accumulate at sites of brain inflammation, and leukocytes (e.g. platelet endothelial cell adhesion molecule-1 (CD31) on monocytes and neutrophils; and (ii) VCAM-1 overexpressed on the membrane has high affinity for very late antigen 4 (VLA-4) on leukocytes. Thus, 4T1 cell membrane-camouflaged bionanoparticles can be exploited to achieve BBB penetration and specific targeting of the ischemic brain parenchyma for IS therapy 178, 179. Based on this, they further designed a bionanodelivery system (MPP/SCB) using 4T1 cell membranes encapsulated with PH-sensitive polymer nanoparticles (PP/SCB) loaded with the antioxidant succinebucil. In a tMCAO rat model, MPP/SCB could be preferentially delivered to the ischemic hemisphere than the normal hemisphere (4.79-fold), producing significant infarct volume reduction (69.9%) and marked neuroprotection compared to the corresponding PP/SCB. These results highlight the translational potential of cancer cell membrane-based bionanoparticles for targeted IS therapy. To advance this platform, future work must address underlying safety concerns through comprehensive profiling of serum tumor markers to preclude oncological risks.

### 4.2. Exosome-involved nanotherapeutics

As biomimetic nanotechnology advances, exosomes, naturally occurring nanoscale extracellular vesicles secreted by cells, have emerged as a highly refined platform for drug delivery and regenerative therapy. While nanoparticles camouflaged with cell membranes can mimic the surface properties of native cells, exosomes offer a more biologically authentic and multifunctional communication system. They possess intrinsic capabilities to traverse biological barriers, evade immune surveillance, and deliver a wide range of biomolecular cargoes to targeted recipient cells. Their endogenous origin, low immunogenicity, and inherent role in intercellular signaling position exosomes as an ideal candidate for addressing the complex pathophysiology of IS. Moreover, exosomes can be engineered to enhance targeting specificity or loading capacity, making them versatile and highly biocompatible carriers for IS therapeutics. This section explores the burgeoning field of exosome-involved nanotherapeutics, covering sources such as MSCs, neuronal cells, immune cells, and even plants. We focus on their dual role as both innate therapeutic agents and engineered delivery systems, and discuss how they are opening new avenues for neuroprotection, immunomodulation, and neural repair in IS.

#### 4.2.1. MSCs-derived exosomes

In the last decade, MSCs therapy has emerged as the most promising candidate in stem cell therapy for IS, but it still faces the following obstacles: (i) poor survival in hypoxic and inflammatory conditions of the brain; (ii) susceptibility to vascular occlusion and reduced cerebral blood flow; and (iii) inefficiency of delivery to ischemic sites in the brain. In addition, safety issues induced by the route of administration, cell delivery time window, and cellular dose are unpredictable 180. MSC-derived exosomes have been shown to mimic the biological functions of MSCs to promote neurological recovery and angiogenesis after stroke, making them an alternative to MSC-based therapy for IS. However, the low brain targeting as drug carriers limits their further application. Recently, intranasal (IN) drug delivery can improve the efficiency of drug delivery to the brain by passing the BBB via olfactory and trigeminal nerves, emerging as a novel delivery modality for MSC-derived extracellular vesicles (MSC EVs) to the brain. Zhou *et al.* developed BDNF-overexpressed MSC-derived cell vesicles (BDNF-MSC EVs) and administered them intranasally to resolve the problem of insufficient endogenous BDNF-induced impaired neurological recovery in IS 181. Their findings revealed that MSC EVs carry substantial quantities of proteins and miRNAs. More importantly, BDNF-MSC EVs significantly increased BDNF protein levels to promote neuronal survival, neurogenesis and synaptic plasticity in the central nervous system (CNS). In addition, compared to MSC EVs, BDNF-MSC EVs contain the five up-regulated miRNAs that modulate immune and inflammatory processes. Therefore, MSC EVs administered intranasally could selectively target the ischemic brain and be taken up by neurons to exert their biological functions, such as anti-inflammation, enhancement of neurogenesis, and angiogenesis in the tMCAO mouse model, which are further enhanced by MSC EVs loaded with BDNF protein 181.

However, the precise mechanism of the intranasal administration is not fully clarified, and there is still a gap between animal models and human clinical practice. Thus, their clinical safety remains to be demonstrated 182. As an alternative approach, Qiu *et al.* demonstrated that intravenous co-administration of tPA with MSC-EVs enhanced their homing to ischemic regions and uptake by astrocytes 183. Moreover, MSC-EVs containing miR-125b-5p can inhibit astrocyte activation and inflammation, thereby preserving BBB integrity and reducing tPA-induced hemorrhagic side effects. To further monitor the distribution of MSC-EVs in the brain after tPA treatment in real time, MSC-EVs were labeled using AIEgens. Compared to commercially available DiR tracer probes, AIEgens provide superior real-time imaging and treatment visualization, owing to their excellent biocompatibility, controlled resistance to photobleaching and high signal-to-noise ratio 183.

#### 4.2.2. Neuronal cell-derived exosomes

Building upon the therapeutic potential of MSC-derived exosomes, attention has turned to exosomes originating from resident neuronal cells, which offer unique advantages due to their innate participation in CNS communication and repair. These exosomes secreted by neuronal cells serve as critical mediators of intercellular signaling, actively regulating neural development, regeneration, and vascular remodeling in the post-stroke brain. For instance, Wang *et al.* identified 176 differentially expressed microRNAs in astrocyte-derived exosomes under ischemic conditions, functionally associated with signal transduction, neuroprotection, and stress adaptation 184. Wu *et al*. demonstrated that miR-32 carried by astrocyte-derived exosomes could attenuate I/R-induced neuronal injury by targeting Toll-like receptor 7 (TLR7) and downregulating the NF-κB/MAPK axis in a rat brain I/R injury model 185. In addition, other neural cell-derived exosomes have been reported to be involved in signaling related to the pathological process of IS, and are increasingly considered as potential nanotherapeutic agents or nanocarriers for IS treatment.

Despite the intimate relationship between neuronal cell-derived exosomes and pathological recovery from IS, there are still some insufficiencies, such as poor brain targeting and rapid in vivo clearance, limiting their clinical application as nanomedicines or nanocarriers. Therefore, an increasing number of researchers have focused on how to efficiently achieve in vivo brain delivery of neuronal cell-derived exosomes. Tian* et al.* developed neural progenitor cell-derived extracellular vesicles (EVsReN) conjugated recombinant protein (RGD-C1C2-EVsReN) to achieve anti-inflammatory therapy 186. Recombinant proteins composed of arginine-glycine-aspartate (RGD)-4C peptide and lactate adhesion protein (C1C2) could rapidly self-associate to membranes upon incubation with EVs. The modified brain-targeting peptide RGDEVsReN exhibited higher brain accumulation and an increased fluorescence ratio (7.84) of the lesion area (ipsilateral) to the nonischemic area (contralateral) compared with unmodified EVsReN 186. Remarkably, the high division capacity of neural progenitor cells (up to 45 generations) enables cost-effective large-scale production.

Neural stem cell (NSC)-derived exosomes promote neurogenesis and migration of NSCs. Similarly, the relatively rapid *in vivo* clearance rate severely limits their therapeutic efficacy. To overcome this problem, Gu *et al.* encapsulated NSC-derived exosomes into a novel injectable catechol-modified hyaluronic acid hydrogel (HAD-Exo) 187. HAD with superior tissue retention properties could adhere to tissue surfaces and release exosomes in a controlled manner. The results showed that PKH26 fluorescently labeled NSC-Exo began migrating to the ischemic site in the mouse brain within 24 h of injection, with fluorescence almost completely disappearing after 14 days. In contrast, HAD-Exo continued to fluoresce around the brain tissues, with detectable fluorescence remaining at 28 days 187. In a tMCAO mouse model, HAD-Exo significantly improved neurological function, promoted cerebral angiogenesis, reduced infarct areas and possessed anti-inflammatory effects 187.

#### 4.2.3. Immunocyte-derived exosomes

**Figure 11:**
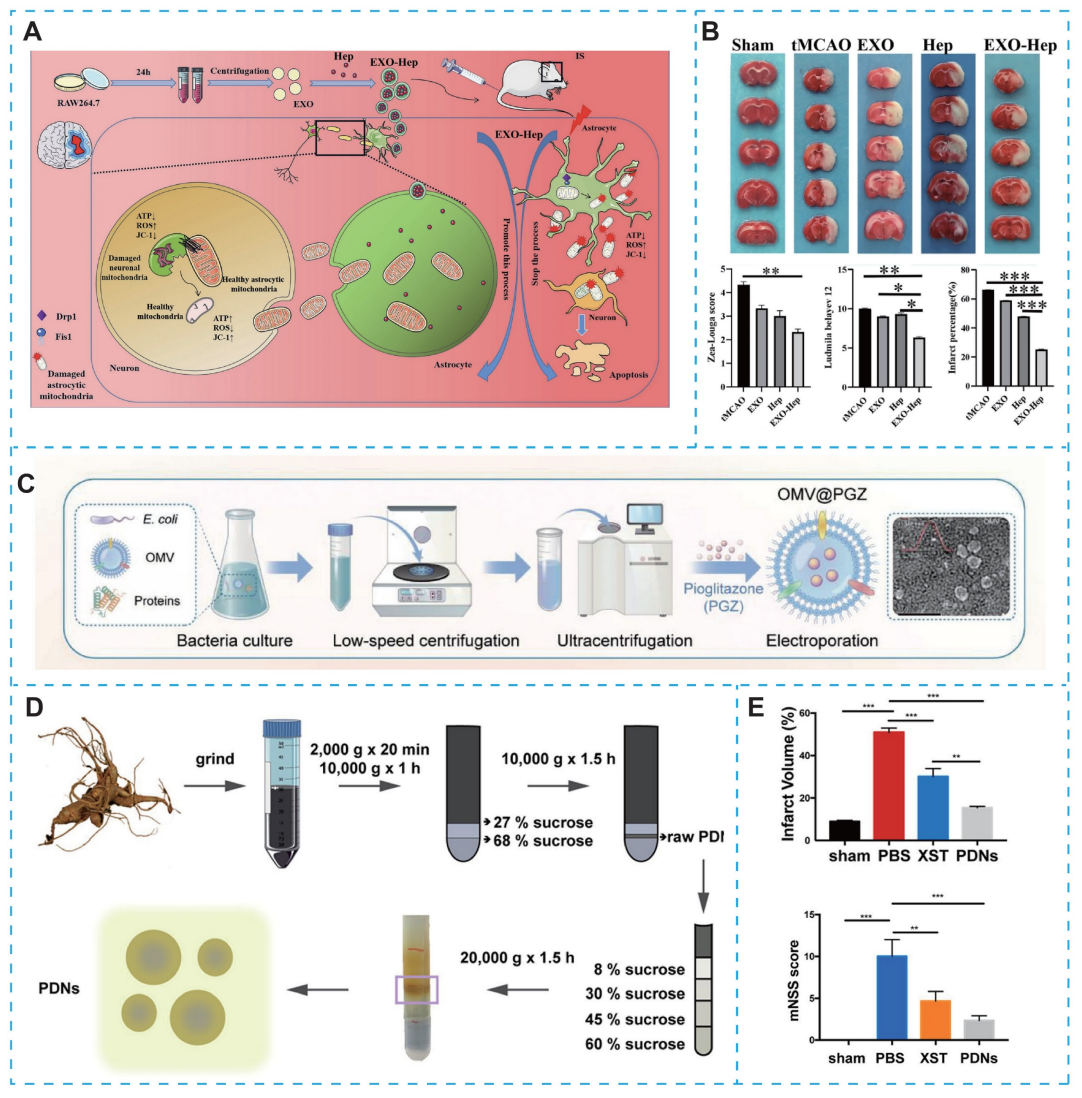
** Exosome-based nano-DDS for IS treatment.** (A) Schematic illustration of Heptapeptide loaded macrophage derived exosomes (EXO-Hep) for the treatment of ischemic stroke. (B) Representative brain slices with infarcts were stained by TTC and infarct volume was calculated in tMCAO rats treated with Hep, EXO and EXO-Hep. Reproduced, with permission, from 189. Copyright (2022), Springer. (C) OMV was extracted by differential centrifugation, and the PGZ was loaded into the OMV by electroporation. Reproduced, with permission, from 190. Copyright (2023), Wiley. (D) Schematic illustration of isolating process of PDNs. (E) Effect of PDNs on CI/R injury in rats. Reproduced, with permission, from 192. Copyright (2023), Springer.

While brain-targeted peptides can enhance exosome accumulation in the brain, their presence of major histocompatibility complex (MHC) molecules and co-stimulatory molecule CD86 on the exosome surface may provoke an immune response, particularly during long-term treatments 188. Fortunately, exosomes derived from immune cells have emerged as promising drug delivery vehicles, especially in brain-targeted therapies. For instance, Yuan *et al*. found that macrophage-derived exosomes (Mφ-Exo) could penetrate the BBB into the inflamed brain without brain-targeting peptide modifications 188. Mφ-Exo were able to penetrate the BBB at a rate 3.1 times faster than normal brain circulation and achieve 5.8-fold higher drug accumulation in ischemic areas, suggesting the Mφ-Exo with intrinsic brain-targeting ability could serve as a promising drug delivery vehicle for IS treatment. This enhanced delivery is due to the interaction of Mφ-Exo with brain microvascular endothelial cells via integrin lymphocyte function-associated antigen 1 (LFA-1), ICAM-1, and carbohydrate-binding C-type lectin receptors, which are upregulated by the inflammatory response in cerebral ischemia, facilitating exosome homing to the ischemic site. Furthermore, Liu *et al*. utilized Mφ-Exo as effective delivery vehicles for heptapeptide (Hep) (**Figure 11A**) 189. Activated astrocytes could lead to mitochondrial dysfunction in neighboring neurons, in turn amplifying neuronal damage after IS. Hep, a Drp1-Fis1 peptide inhibitor P110, with the ability to attenuate mitochondrial dysfunction in astrocytes, could promote healthy mitochondria of astrocytes into neighboring neurons and ameliorate neuronal mitochondrial function to reduce neuronal damage 189. When delivered by Mφ-Exo, Hep overcame the obstacles such as enzymatic degradation, short half-life and poor membrane permeability. More importantly, Mφ-Exo efficiently delivered Hep to the ischemic brain regions, enhancing drug distribution in activated astrocytes and significantly reducing cerebral ischemia-reperfusion injury in a tMCAO model (**Figure 11B**). Overall, immunocyte-derived exosomes represent an advanced delivery platform that combines intrinsic brain-targeting capability with reduced immunogenicity, demonstrating particular utility for delivering protein macromolecule drugs in IS therapeutics.

#### 4.2.4. Other exosomes

Bacterial-derived outer membrane vesicles contain lipopolysaccharide (LPS), which can be specifically recognized by neutrophil Toll-like receptor to promote endocytosis of the vesicles by neutrophils. Neutrophils, as the first cells activated and recruited from the periphery to the ischemic brain, actively move toward the endothelial junction through interactions with endothelial cells by rolling, adhering, and crawling, and eventually migrate to the BBB. Therefore, a decoy nanoparticle with bacterial-derived outer membrane vesicles to specifically piggyback on neutrophils *in situ* in the blood circulation and migrate toward ischemic foci using neutrophils *in vivo* as an anchoring target has also emerged as a promising nanodelivery strategy. Pan *et al*. constructed a bacterial-derived outer membrane vesicle encapsulated with pioglitazone (PGZ) (OMV@PGZ) (**Figure 11C**) 190. Due to the inherited functions associated with bacterial outer vesicles, OMV@PGZ became an ideal bait for neutrophil uptake, with the majority of nanoparticles being phagocytosed within 90 minutes of incubation. OMV@PGZ then penetrated the BBB into the infarcted brain by attaching to neutrophils. Once in the ischemic zone, neutrophils undergo NETosis triggered by excessive ROS, releasing PGZ upon neutrophil disintegration. *In vivo* experiments showed that OMV@PGZ alleviated reperfusion injury and exerted neuroprotective effects by inhibiting the activation of the NLRP3 inflammasome and ferroptosis. Additionally, their study identified the involvement of the transcription factors Pou2f1 and Nrf1 in oligodendrocytes, which promoted neural repair as revealed by single-nucleus RNA sequencing (snRNA-seq) 190.

Exosomes from different cells (including MSCs, neural cells, and immune cells) have now been shown to be beneficial for IS recovery and have become promising nanodelivery platforms by virtue of their natural carrier advantage and ability to cross the BBB. However, cell-derived exosomes have low yields and typically take 3-4 days to obtain under laboratory conditions. Surprisingly, exosomes could also be isolated from fresh plant tissues and their large-scale production could be achieved in one day. Obviously, plant-derived exosomes offer superior scalability compared to cell-derived exosomes, attracting growing interest in their therapeutic potential for IS. Huang et al. found that exosomes from bitter melon (Momordica charantia) (MC-sEVs) can alleviate stroke-induced neuronal ferroptosis by inhibiting the ubiquitination and degradation of glutathione peroxidase 4 (GPX4), thus promoting neurological recovery 191. Additionally, some natural medicines, with their multi-component, multi-target profiles and low toxicity, have garnered significant interest for their potential in preventing and treating cerebral infarction and ischemia-reperfusion injury.

Panax notoginseng (PN) is one of the most commonly used traditional Chinese medicines for the treatment of IS. Based on this, Li *et al.* investigated the potential role of PN-derived exosome-like nanoparticles (PDN) in IS treatment (**Figure 11D**) 192. They demonstrated that PDNs could cross the BBB and were effectively internalized by microglia. PDNs inhibited microglia-induced inflammation and attenuated CI/R damage by transforming M1 microglial cells into the M2 subtype, and they verified that lipids in PDNs were the main active components of anti-inflammatory effects (**Figure 11E**) 192. Similarly, Zhang et al. systematically studied the neuroprotective benefits of exosomes derived from Houttuynia cordata in cerebral ischemia/reperfusion injury. They found that these exosomes are rich in miR159a, effectively reducing neuronal apoptosis and ferroptosis, and promoting recovery of neuronal function 193. More importantly, Wang *et al.* discovered that plant-derived matrine exosome-like nanovesicles (MC-ELNs) can protect the integrity of the BBB in stroke rats and alleviate tPA-induced hemorrhage by suppressing the expression of MMP-9 194.

Although plant-derived exosome-like nanoparticles hold great promise due to their biocompatibility, inherent anti-inflammatory and antioxidant properties, and ability to cross the BBB, making them ideal as both therapeutic agents and delivery vehicles. However, they still face numerous challenges, including the incomplete understanding of the complex active ingredients and mechanisms of action, difficulties in standardizing preparation processes and quality control, and limitations in the natural targeting efficiency 195, 196. Future research should leverage multi-omics technologies and surface engineering strategies to elucidate molecular mechanisms, achieve standardized production, and optimize targeting efficiency, thereby advancing the clinical translation of green therapeutic strategies.

## 5. Discussion

In recent years, nano-DDS have emerged as a transformative strategy to overcome the formidable challenges in IS therapy, including the narrow therapeutic window of thrombolytics, the impermeability of the BBB, and the complex pathophysiology involving excitotoxicity, oxidative stress, and neuroinflammation. This review has systematically chronicled the evolution of nano-DDS from conventional platforms to cutting-edge biomimetic and biological systems. While significant progress has been made in enhancing drug bioavailability, targeting efficiency, and therapeutic outcomes in preclinical models, each platform possesses a unique set of advantages and inherent limitations that dictate its clinical translation potential. **Table 4** provides a comparative summary of the key nano-DDS platforms discussed, delineating their core advantages, primary limitations, and representative studies.

Obviously, there is no universal nano-DDS for IS. The choice of platform must be strategically aligned with the therapeutic objective and the temporal stage of the disease. For instance, in the hyperacute phase, the priority is rapid and precise thrombolysis. In the hyperacute phase, the primary therapeutic goal is rapid and precise thrombolysis. Platelet-membrane camouflaged nanoparticles and thrombin-responsive self-assembled nanomotors exhibit enhanced targeting specificity to the thrombus, providing significant therapeutic potential. However, their efficacy in subsequent neuroprotection remains limited, unless co-delivered with neuroprotective agents. In contrast, during the subacute phase, characterized by neuroinflammation and oxidative stress, macrophage membrane-coated nanozymes and exosomes emerge as more promising candidates. These nanoplatforms possess the intrinsic ability to home to inflamed brain regions, exerting direct modulatory effects on the immune microenvironment, thus offering a more robust therapeutic strategy. Notably, conventional polymeric nanoparticles and liposomes, while less "smart" than biomimetic systems, benefit from decades of formulation experience and established regulatory pathways, giving them a shorter path to clinical application. In contrast, the most advanced platforms like exosomes and cell membrane-camouflaged nanoparticles, despite their superior biological performance, face monumental challenges in scalable manufacturing, quality control, and ensuring batch-to-batch consistency, which currently represent the primary barriers to their clinical adoption.

**Figure 12:**
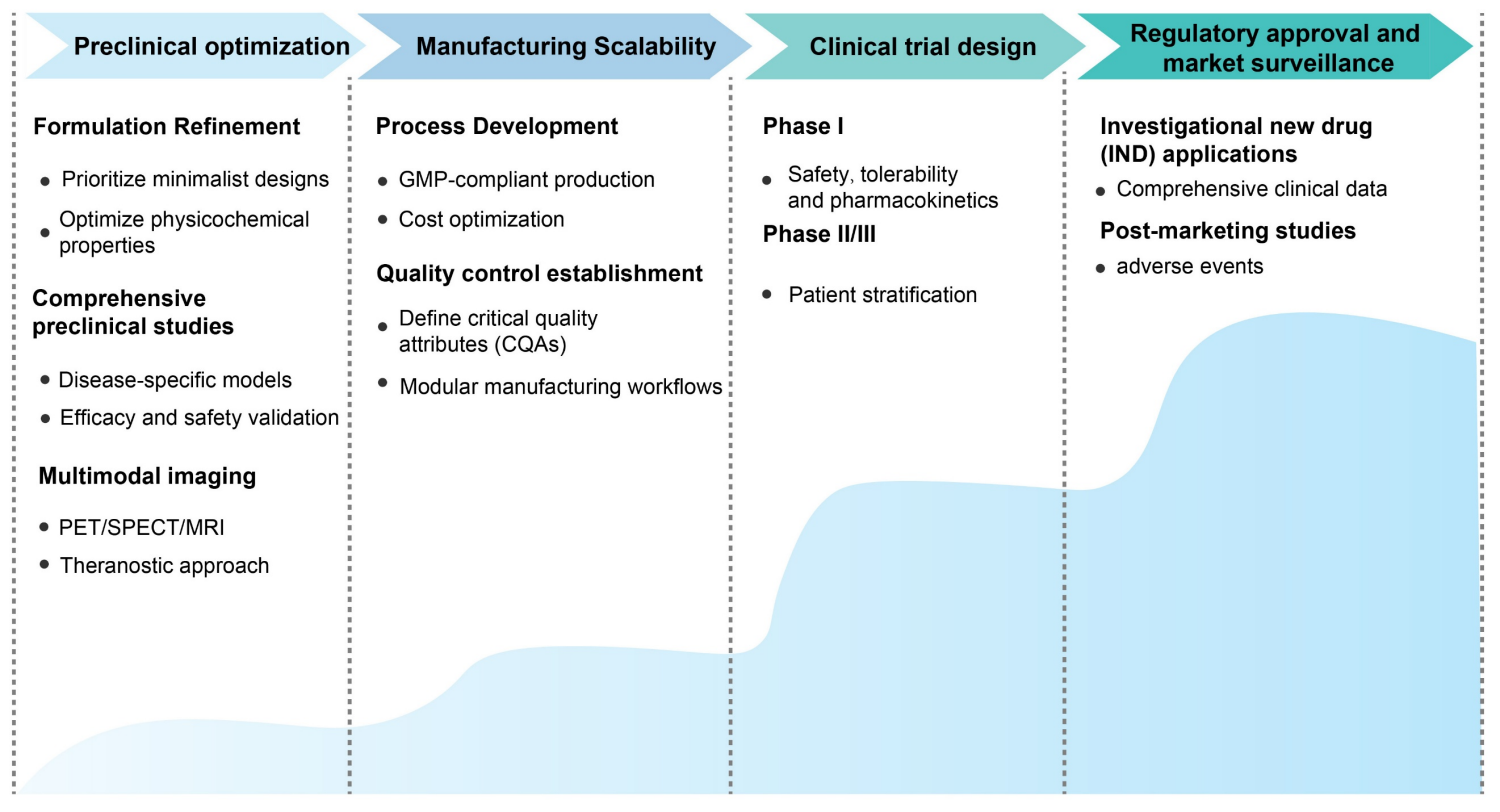
Clinical translation roadmap for nano-DDS in IS, integrating preclinical optimization, manufacturing scalability, regulatory compliance, and clinical validation.

To bridge the gap between preclinical promise and clinical reality, a systematic and phased approach is imperative, focusing on the following critical components (**Figure 12**): (i) Preclinical optimization. Focus on minimalist designs, such as self-assembled small-molecule prodrugs, to enhance manufacturability and minimize batch-to-batch variability. Optimize physicochemical properties (size, surface charge, targeting ligand density) to balance BBB penetration, circulation time, and target specificity. Conduct comprehensive preclinical studies using disease-specific models to confirm nano-DDS efficacy in reducing infarct volume, improving neurological function, and mitigating ischemia-reperfusion injury. More importantly, utilize multimodal imaging to dynamically monitor the spatiotemporal distribution of nano-DDS, verifying successful BBB penetration and target engagement, thereby enabling a theranostic approach that links diagnostic readouts to therapeutic efficacy. (ii) Manufacturing scalability. Transition from laboratory-scale synthesis to GMP-compliant production. Minimize production costs by selecting low-cost raw materials and simplifying synthesis steps. Define critical quality attributes (CQAs) including particle size distribution, drug loading efficiency, release kinetics, and purity. Additionally, for biomimetic systems (e.g., cell membrane-camouflaged nanoparticles and exosomes), standardize cell source selection, membrane isolation, and coating processes to maintain consistent biological activity and develop modular manufacturing workflows to reduce customization complexity. (iii) Clinical trial design. Design phase I trials to evaluate safety, tolerability, and pharmacokinetics in healthy volunteers or IS patients. Phase II/III trials should focus on patient stratification (e.g., based on BBB permeability biomarkers) to enroll patients most likely to benefit, with primary endpoints including neurological function improvement (e.g., NIHSS score) and secondary endpoints such as infarct volume reduction and bleeding risk. (iv) Regulatory approval and market surveillance. For novel nanomaterials, compile comprehensive clinical data on pharmacokinetics, toxicity, and mechanism of action to support investigational new drug (IND) applications. Safety remains a paramount concern for nano-DDS, particularly given the vulnerability of the ischemic brain and potential long-term effects of nanomaterial accumulation. Therefore, post-marketing studies should track adverse events (e.g., bleeding complications, chronic inflammation, organ toxicity) and assess efficacy across diverse patient populations (e.g., elderly patients or those with comorbidities). Finally, the personalized treatment plan should be finalized based on the collected clinical data. In summary, clinical translation will depend on addressing biosafety, scalability, and regulatory challenges. Future research should focus on developing standardized evaluation frameworks and long-term toxicity studies to ensure safe clinical application.

## 6. Conclusions and outlook

In conclusion, nano-DDS represent a transformative approach to IS treatment, offering solutions to longstanding challenges in drug delivery and therapeutic efficacy. Looking ahead, the incorporation of real-time imaging tools with therapeutic nanoparticles could enable precise monitoring of drug distribution and treatment response, facilitating dynamic, patient-specific therapeutic adjustments. The integration of advanced technologies such as artificial intelligence (AI) and machine learning (ML) could revolutionize the design and optimization of nano-DDS. AI-driven algorithms can aid in predicting drug release kinetics, optimizing nanoparticle formulations, and identifying novel targeting ligands. Additionally, the combination of nano-DDS with emerging therapies, such as gene editing and stem cell therapy, holds immense potential for synergistic effects in stroke treatment. For instance, CRISPR-Cas9-loaded nanoparticles could be used to target specific genes involved in stroke pathology, while stem cell-derived exosomes could enhance tissue repair and regeneration. By leveraging interdisciplinary collaborations and cutting-edge technologies, the next generation of nano-DDS could pave the way for more effective, personalized, and accessible treatments for IS.

## Figures and Tables

**Table 1:** A summary of liposome and polymeric nanoparticles for IS treatment.

Conventional carrier material	Drug loading	Mechanism	Refs
Liposome	Edaravone	Antioxidant and anti-inflammatory	86
	Ginkgolide B	Antioxidant and anti-inflammatory	87
	Melatonin	Antioxidant and anti-inflammatory	88
	Compound 5-(3-Bromophenyl)-1,3-dihydro-2H-Benzofuro[3,2-e]-1,4-diazepin2-one (5BDBD)	Neuroprotective effect Fluorescent tracking	89
	Emodin	Brain targeting capabilities conferred by cyclic Arg-Gly-Asp (cRGD) peptide Neuroprotective effect and anti-inflammatory	90
	Xenon (Xe)	Neuroprotective effects triggered by non-toxic gases Timely ultrasound imaging	91
Polymer	Idebenone	CREKA peptide-mediated thrombus targeting ROS-responsive drug release Inhibition of neuronal iron death and anti-inflammatory	92
	Curcumin	AIEgens-driven lesion site-specific fluorescence turn-on and drug release monitoring Antioxidation, antiapoptosis, and anti-inflammatory	93
	Resveratrol	pH-responsive drug release cRGD-mediated brain targeting Triphenylphosphine (TPP)-driven mitochondrial targeting antioxidant and anti-inflammatory	94
	Urokinase-type Plasminogen activator (uPA) and superoxide dismutase (SOD)	pH-responsive drug release Microvessel thrombolysis Antioxidant and anti-inflammatory	95
	Glabridin	Spleen Targeting Neuroprotective effect and anti-inflammatory	96
	Baicalin	Brain targeting capabilities conferred by RVG29 peptide Neuroprotective effect and anti-inflammatory	97
	Alpha-mangostin (α-M)	α-M/albumin complex may cross the BBB through the absorptive-mediated transcytosis pathway Neuroprotective effect and anti-inflammatory	98
	Bendavia (SS-31) and Puerarin (PU)	ROS-responsive drug release SS-31-mediated mitochondrial targeting and antioxidant effects Neuroprotective effect and anti-inflammatory	99
	6-bromoindirubin-3′- oxime (BIO) and vascular endothelial growth factor (VEGF)	Sequential drug release Rapid anti-inflammatory and sustained angiogenic effects	65
	Curcumin and edaravone	Sustained drug release for two weeks Antioxidant and anti-inflammatory	100
	Heme oxygenase-1 (HO1) self-Replicating mRNA	Long-term antioxidant and anti-inflammatory	101
	Ginkgolide B	Fluorescent L-aspartic acid-driven brain targeting Fluorescence monitoring Antioxidant and anti-inflammatory	102
	Glyburide	ROS-responsive drug release AMD3100-mediated brain targeting PTT carrier-driven antioxidant effects Glyburide-driven anti-edema	103

**Table 2:** A summary of Molecularly self-assembled nanoparticles and Nanozymes for IS treatment.

	Polymers	Drugs/enzyme-like substances	Mechanisms	Refs
Polymeric prodrug nanoassemblies	Polyethylene glycol (PEG) and poly (ethylene glycol)-block-poly (propylene glycol)-block-poly (ethylene glycol) (PEG-PPG-PEG, a thermosensitive polymer)	Urokinase plasminogen activator (uPA)	pH/temperature-responsive drug release Thrombolytic therapy Therapeutic hypothermia	137
	Chitosan	Myricetin	ROS-responsive drug release Antioxidant and anti-inflammatory	138
	Hyaluronic acid	Rutin	pH-responsive drug release SS-31-mediated BBB penetration and mitochondrial targeting Neuroprotective effect and anti-inflammatory	139
	Diethylaminoethylen (DEAE)-dextran	18β-glycyrrhetinic acid (GA)	ROS-responsive drug release Anti-inflammatory	140
Peptide/gene drug nanoassemblies	-	Tetrahedral framework nucleic acid	Neuroprotective effect and anti-inflammatory	141
Nanozymes	-	Two dimensional (2D) vanadium carbide (V_2_C) MXene	Antioxidant and anti-inflammatory	142
	-	Iron oxide	Neuroprotective effect and anti-inflammatory Prevent the injury of neurons and blood-brain barrier integrity from ischemic stroke	143
	-	Cerium (Ce)-doped Linde Type A (LTA) zeolite	Neuroprotective effect and anti-inflammatory	144
	-	Cerium oxide and dl-3-n-butylphthalide	Nanozymes as carriers for dl-3-n-butylphthalide Antioxidant and neurovascular repair abilities	145
	-	Semiconductor material Cu_2_O coated Au heterostructure	Significant antioxidant capacity	146
	-	Peptide-templated MnO_2_	Thrombin-responsive drug release CREKA peptide-mediated thrombus targeting Transferrin-mediated brain targeting Thrombolysis and neuroprotective effect	147
	-	MnO_2_	Nanozymes as carriers for Ginkgolide B (GB) Thrombus Inhibition Neuroprotective effect and anti-inflammatory	148
	-	MnO_2_	Transferrin-mediated brain targeting Nanozymes as carriers for edaravone Mn^2+^ ions-mediated magnetic resonance imaging Neuroprotective effect and anti-inflammatory	149

**Table 3:** A summary of cytomembrane-camouflaged biomimetic nanomedicines for IS treatment.

Cell membrane	Drug/nano-drug	Mechanisms	Route	Refs
Erythrocyte membrane	Janus-type polymeric micromotors (JPMs) composed of heparin (Hep) and chitosan (CHI)	Erythrocyte membrane-endowed stealth properties Photothermal effect-driven nanomotor thrombolysis	-	150
	Fullerenols-loaded mesoporous silica nanoparticles	Erythrocyte membrane-endowed stealth properties Fullerenols-driven thrombolysis	-	151
Platelet membrane	miRNA-Let-7c- loaded pH-sensitive polymeric nanoparticles	Platelet membrane-endowed stealth properties Platelet membrane-mediated BBB penetration and damaged blood vessels targeting pH-responsive drug release Regulation of neuronal apoptosis and microglia phenotypic transformation by miRNA-Let-7c/Notch1 signaling pathway	I.V.	152
	L-arginine and γ-Fe_2_O_3_ magnetic nanoparticles	γ-Fe_2_O_3_ nanoparticles- mediated Localization of stroke lesions and assessment of the severity of the damaged vascular network by multiparameter MRI including T2 * -weighted imaging (T2 * WI) and diffusion-weighted imaging (DWI) Platelet membrane with the ability to target damaged vessels improve classification and precise localization of stroke lesions detected by MRI L-arginine-mediated vasodilation and restoration of blood supply in ischemic lesions	I.P. and I.V.	153
	Human fat extract (FE)-encapsulated PLGA	RGD peptides-modified platelet membrane with the ability to actively target ischemic stroke regions and increase circulation time Sustained drug release triggered by degradability of PLGAs FE containing neurotrophic factors such as BDNF, GDNF and bFGF promotes angiogenesis and neurobehavioral recovery	I.V.	154
	Deoxyribonuclease I (DNase I)-loaded hollow Prussian blue nanoparticle	Salic acid (SA)-modified platelet membrane with the ability to actively hitchhike on neutrophils into the injured brain parenchyma Significant antioxidant capacity	I.V.	155
Neutrophil membrane	Edaravone-encapsulated RCD (phenylboronic acid pinacol ester (PBAP)-modified β-CD) nanoparticles	SHp peptides-modified neutrophil membrane with the ability to target inflammatory sites and injured neurons PBAP-mediated ROS-responsive drug release and ROS consumption Edaravone-driven inflammatory inhibition and neuroprotection	I.V.	156
	Leonurine (Leo)-encapsulated liposomes	Neutrophil membrane-mediated inflammatory sites targeting Leo-mediated antioxidation, anti-inflammation and neuroprotection High affinity between neutrophil membranes and inflammatory factors realizes synergistic anti-inflammation with Leo	I.V.	157
	Nitric oxide donor hydroxyurea (HYD) and edaravone-loaded hypoxia-sensitive liposomes	Neutrophil membrane-mediated inflammatory sites targeting HYD-induced enhancement of blood-brain barrier permeability and local cerebral blood flow promotes nanoparticle transport into the penumbra 2-Nitroimidazole-based hypoxia-responsive drug release Edaravone-driven inflammatory inhibition and neuroprotection	I.V.	158
	FTY720-loaded polyprodrug nanoparticles	Neutrophil membrane-enhanced BBB penetration and inflammatory sites targeting ROS-responsive drug release FTY720 promotes M2 polarization of microglia and inflammation suppression	I.V.	159
Macrophage membrane	Baicalin-loaded liposomes	Macrophage membrane-enhanced BBB penetration and brain targeting Baicalin-mediated antioxidation, anti-inflammation and neuroprotection	I.V.	160
M2 phenotype microglia membrane	Nanovehicles (FP) composed of polyfluorocarbon and Pluronic P123	M2 phenotype microglia membrane-mediated inflammatory sites targeting Polyfluorocarbon delivers oxygen to alleviate the hypoxic environment of ischemic brain Pluronic P123 inhibits matrix metalloproteinase-9 to protect BBB	I.V.	161
neural stem cell membrane	Metformin-loaded liposomes	Neural stem cell membrane targets the injured brain microvessel endothelial cells (BMECs) and penetrate into the lesion through the vascular cell adhesion molecule-1 (VCAM-1)/VLA-4 interaction Metformin-mediated anti-inflammation and the recovery of BBB	I.V.	162

**Table 4:** Comparative analysis of nano-DDS platforms for IS therapy.

Nano-DDS Platform	Key advantages	Major limitations
Liposomes and polymeric nanoparticles	• Well-established synthesis and characterization • Tunable drug release kinetics (e.g., pH, ROS-responsive) • Good biocompatibility with FDA-approved materials (e.g., PLGA)	• Rapid clearance by RES without PEGylation or targeting ligands • Potential carrier-related toxicity or immunogenicity • Relatively low targeting specificity compared to biomimetic systems • Batch-to-batch variability
Inorganic nanoparticles	• Unique intrinsic therapeutic properties (e.g., antioxidant nanozymes) • Superior physical stability • Facile functionalization of surface • Capabilities for multi-modal imaging (e.g., MRI, fluorescence)	• Potential long-term toxicity and bioaccumulation of metal components • Unpredictable biodegradation and potential inflammatory responses • Limited targeting ability without surface functionalization
Carrier-free nanoassemblies	• Ultrahigh drug loading capacity • No excipient-induced toxicity• Simplified preparation process• Defined molecular structure	• Limited to drugs with self-assembly properties• Potential stability issues in complex biological fluids• Challenging to control the size and polydispersity
Cell membrane-camouflaged nanomedicines	• Superior biocompatibility and prolonged circulation• Intrinsic targeting to thrombus (platelets) or inflamed endothelium (immunocytes)• Effective evasion of the immune system (e.g., RBC membrane)• Multi-functional synergy	• Complex and time-consuming preparation process• Batch-to-batch variability due to biological source• Risk of pathogen contamination• Limited availability of source cells for large-scale production
Exosome-based nanomedicines	• Naturally low immunogenicity• Innate ability to cross biological barriers, including the BBB• Innate tropism to injured tissues (e.g., MSC-EVs to inflammation)• Function as both a carrier and a therapeutic agent	• Extremely low production yield and high cost• Difficulties in standardization, isolation, and drug loading• Heterogeneity in size and cargo• Incomplete understanding of their *in vivo* fate and mechanism

## Abbreviations

IS: Ischemic stroke
tPA: tissue plasminogen activator
RONS: reactive oxygen species and nitrogen species
BBB: blood-brain barrier
nano-DDS: nanodrug delivery systems
NPs: nanoparticles
NMDA: N-methyl-D-aspartate
CaMKII: calcium/calmodulin-dependent protein kinase II
CaM: calmodulin
AMPA: α-amino-3-hydroxy-5-methyl-4-isoxazolepropionic acid
nAChR: nicotinic acetylcholine receptor
MMPs: matrix-degrading enzymes
VCAM-1: vascular cell adhesion molecule 1
CB1: cannabinoid 1
GPIIb/IIIa: glycoprotein IIb/IIIa
GPIbα: glycoprotein Ibα
ROS: reactive oxygen species
MMP-9: matrix metalloproteinase-9
PAD4: peptidylarginine deiminase 4
TK: thioketone
NETs: neutrophil extracellular traps
TJ: tight junctions
PLGA: poly (lactic-co-glycolic acid)
PAMAM: polyamidoamine
uPA: urokinase plasminogen activator
PTT: photothermal therapy
ICG: indocyanine green
SHp: stroke-homing peptide
AR: aspect ratio
PEG: polyethylene glycol
PCL: poly-ε-caprolactone
NGF: nerve growth factor
PBAP: phenylboronic acid pinacol ester
TfR: transferrin receptor
RES: reticuloendothelial system
RBC: red blood cell
ICAM-1: intercellular adhesion molecule-1
CNS: central nervous system
CAT: acid-responsive catalase
MSC: mesenchymal stem cell
NF-κB: nuclear factor kappa-B
SIRPα: signal regulatory protein α
cGAS-STING: cyclic GMP-AMP synthase-stimulator of interferon genes
CREKA: Cys-Arg-Glu-Lys-Ala
TK: thioketone
